# In Silico Drug Repositioning Identifies SYK Kinase Inhibitors as Potential Neuroprotective Agents for Ataxia–Telangiectasia

**DOI:** 10.1111/cbdd.70354

**Published:** 2026-07-01

**Authors:** Alessia Romano, Rocco Buccheri, Chiara Zagni, Antonio Rescifina

**Affiliations:** ^1^ Department of Drug and Health Sciences University of Catania Catania Italy

**Keywords:** Ataxia–Telangiectasia, drug repositioning, molecular docking, molecular dynamics, network medicine, structure‐based drug design, SYK kinase

## Abstract

Ataxia–Telangiectasia (AT) is a rare neurodegenerative disorder characterized by progressive neuronal loss and chronic neuroinflammation. Emerging evidence indicates that aberrant overexpression of the adaptor protein TYROBP promotes sustained recruitment and activation of spleen tyrosine kinase (SYK), contributing to pathogenic inflammatory signaling in AT. In this study, we applied a drug repositioning strategy to identify clinically approved compounds that inhibit SYK activity. An integrated *in silico* workflow was employed, combining convolutional neural network (CNN)–based molecular docking with network medicine analysis using the SAveRUNNER platform to screen a library of 2342 FDA‐approved drugs. The top‐ranked candidates were further evaluated using long‐timescale molecular dynamics simulations and post‐simulation redocking to assess their binding stability and conformational persistence within the SYK catalytic pocket under explicit solvent conditions. This multilevel computational analysis identified vemurafenib and palbociclib as the most promising SYK inhibitors, both exhibiting sustained binding affinity and stable intermolecular interactions following protein relaxation. These findings support the feasibility of repurposing FDA‐approved drugs to modulate SYK‐driven neuroinflammatory pathways and provide a mechanistic framework for developing novel neuroprotective interventions for AT.

## Graphical abstract

Repositioning of vemurafenib and palbociclib as spleen tyrosine kinase inhibitors for the treatment of neuroinflammation in Ataxia–Telangiectasia.

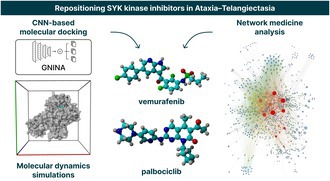

**Abbreviations**
AT: Ataxia‐Telangiectasia
ATM: Ataxia Telangiectasia mutated
CCAS: cerebellar cognitive affective syndrome
CNNs: convolutional neural networks
CNS: central nervous system
DDR: DNA damage response
DSBs: double‐strand breaks
FDA: Food and Drug Administration
FEL: free energy landscape
HTVS: high‐throughput virtual screening
ITAMs: immunoreceptor tyrosine‐based activation motifs
*K*_d_
: dissociation constant
MC: Monte Carlo
MD: molecular dynamics
NK: natural killer
PCA: principal component analysis
RMSD: root mean square deviation
SASA: solvent accessible surface area
SH2: src homology‐2
SYK: spleen tyrosine kinase
TYROBP: tyrosine kinase‐binding protein
VS: virtual screening

## 1 Introduction

Ataxia‐Telangiectasia (AT), also known as Louis‐Bar syndrome, is a rare autosomal recessive genetic disorder caused by mutations in the Ataxia Telangiectasia Mutated (ATM) gene, which encodes the homonymous protein (Gumy‐Pause et al. 2004). ATM plays a key role in the cellular response to DNA damage, particularly in the repair of double‐strand breaks (DSBs). AT is often described as a genomic instability syndrome, chromosomal instability disorder, DNA repair defect, or DNA damage response (DDR) syndrome (Shibata and Jeggo 2021). Moreover, ATM is highly expressed in Purkinje cells of the cerebellum, neurons, and endothelial cells of the skin and mucosa. AT is characterized by severe combined immunodeficiency (particularly affecting antibody responses) and progressive cerebellar ataxia. The estimated incidence is approximately one case per 100,000 children. The clinical presentation is heterogeneous, and the severity of neurological, respiratory, and immunological manifestations varies widely among patients (Rothblum‐Oviatt et al. 2016). Neurological symptoms usually appear between the first and second years of life, beginning with postural instability, loss of balance, and abnormal head movements. As the disease progresses, tremors and worsening motor coordination deficits develop, with many patients requiring wheelchairs by late childhood. Cognitive deficits in AT appear early, in parallel with cerebellar involvement, and exhibit characteristics typical of cerebellar cognitive affective syndrome (CCAS). In advanced disease stages, as neurological damage extends beyond the cerebellum, these cognitive impairments become broader and more pronounced (Hoche et al. 2014). The prognosis is generally poor, with approximately 35% of patients developing cancer before the age of 20.

At the cellular level, ATM deficiency results in marked radiosensitivity to ionizing radiation, chromosomal instability, telomere shortening, and premature senescence, reflecting a defective response to DNA double‐strand breaks (DSBs), which are critical for maintaining genomic stability. Loss of ATM function leads to the accumulation of oxidative damage, disruption of cell cycle checkpoints, and acceleration of neurodegenerative processes.

In addition to the well‐known dysfunction of the central nervous system (CNS), recent evidence suggests that neuroinflammation is actively involved in disease progression. In particular, microglial cells lacking functional ATM exhibit intrinsic activation and enhanced pro‐inflammatory signaling that precedes neuronal apoptosis. Studies using AT‐patient‐derived induced pluripotent stem cells (iPSCs) have demonstrated that activated microglia induce cytotoxic effects when co‐cultured with cerebellar neurons, supporting the direct contribution of microglial activation to Purkinje and granule neuron degeneration (Lai et al. 2024).

Among the molecular mediators of this inflammatory response, TYRO protein tyrosine kinase‐binding protein (TYROBP) is significantly overexpressed in patients with AT. It plays a crucial role in regulating inflammatory responses in the brain. In its tyrosine‐phosphorylated form, TYROBP can activate an intracellular cascade through the recruitment of spleen tyrosine kinase (SYK), which activates effector molecules involved in the release of pro‐inflammatory cytokines, such as TNF‐α and IL‐1β. Persistent microglial activation can exacerbate neuronal damage, as overactive microglia release neurotoxic factors, including reactive oxygen species (ROS), nitric oxide, and excitatory amino acids, thereby amplifying neurodegeneration (Zhou et al. 2024).

These alterations underscore the essential role of ATM protein in genome surveillance and DNA repair. When ATM is nonfunctional or absent, oxidative damage accumulates, cell cycle checkpoints are impaired, and neurodegenerative processes are accelerated. Given that the neuroinflammatory microenvironment and DNA damage pathways converge in the pathogenesis of AT, understanding the cellular alterations associated with the disease is essential for properly framing the potential role of TYROBP and guiding future therapeutic strategies based on selective inhibition of its downstream signaling. To date, no definitive cure for AT exists. Treatment remains primarily focused on multidisciplinary clinical management, including the control of immunodeficiency, pulmonary infections, neurological dysfunctions, and associated neoplasms. Neurorehabilitation also plays a crucial role, involving physiotherapy, occupational therapy, and speech therapy focused on swallowing, as well as the use of adaptive aids and personalized nutritional support (Perlman et al. 2003).

In this context, the TYROBP‐SYK signaling axis has emerged as a critical mediator of neuroinflammation driven by microglia. TYROBP (also known as DAP12) is a transmembrane adaptor protein containing an ITAM motif in its cytoplasmic domain. Although TYROBP does not transmit signals, it forms complexes with surface receptors such as TREM2, SIGLEC‐15, and SIRPβ, and is essential for intracellular signal transduction. When these receptors are activated, the ITAM motif of TYROBP is phosphorylated by a protein kinase (such as SRC), creating a docking site for SYK, which binds to the phosphorylated ITAM and is thus activated. SYK activation triggers an intracellular signaling cascade that promotes microglial activation, production of pro‐inflammatory cytokines (e.g., IL‐1β and TNF‐α), phagocytosis, and other immune responses (Haure‐Mirande et al. 2022). In the brains of patients with AT, TYROBP is upregulated, often in association with activated microglia (Sunila et al. 2024). This suggests that the TYROBP–SYK axis is hyperactive, contributing to chronic neuroinflammation. Persistent neuroinflammation is a key contributor to neuronal damage in AT, particularly in cerebellar Purkinje cell degeneration.

Given the central role of SYK kinase in this pathogenic signaling pathway, its targeted inhibition represents a promising therapeutic strategy for attenuating the neurodegenerative component of AT. The present study aimed to identify small molecules that inhibit SYK using an integrated computational approach, thereby modulating the TYROBP–SYK inflammatory axis and providing a rationale for drug repositioning strategies for AT.

The methodology adopted involved the coordination of various *in silico* techniques, ranging from high‐throughput virtual screening (HTVS) to the analysis of molecular interactions via docking and molecular dynamics (MD) simulations, as well as *in silico* predictions based on the human interactome. This workflow enables the filtering of vast compound libraries, focusing on molecules with known safety profiles. The investigation led to the identification of two FDA‐approved drugs, vemurafenib and palbociclib, which show a high theoretical affinity for the SYK catalytic site and a potential efficacy profile in modulating the TYROBP–SYK inflammatory pathway. The identification of molecules already in clinical use paves the way for a drug repositioning strategy, a crucial approach in the context of rare diseases such as AT, as it has the potential to drastically reduce the time and cost required for the clinical translation of new neuroprotective therapies.

## 2 Materials and Methods

### 2.1 Protein Preparation

The protein was prepared using YASARA software (v. 25.1.13, YASARA Biosciences GmbH, Vienna, Austria) by adding hydrogens using the “Clean → All” option. Finally, the co‐crystallized ligand and water molecules were removed using YASARA software.

### 2.2 Dataset and 3D Structures Generation

The FDA‐approved drug dataset was provided by MCE (MedChemExpress) in the SDF file format. The SDF file was cleaned in DataWarrior by removing the salts and duplicate molecules. Ligand preparation was performed using Open Babel (v. 3.1.1) (O'Boyle et al. 2011), where 3D structures were generated and minimized at physiological pH (7.4). The 3D structures were then optimized at the GFN2 semiempirical level using the xTB (extended tight‐binding, v. 6.7.1) package (Bannwarth et al. 2021). Geometry optimization was performed using an analytical linearized Poisson‐Boltzmann (ALPB) model for water, with charge states assigned to each molecule based on the physiological pH.

### 2.3 Molecular Docking Analysis

Molecular docking analysis was performed using the molecular docking algorithm GNINA (v. 1.3) (McNutt et al. 2025). Regarding GNINA, we used the “rescore” docking mode, which involved 15 ligand pose rotations, and the poses were sorted by the CNN score. The protein input was in the PDB format, and the ligand input was in the SDF format. The simulation box was built using AutoDock Tools (v. 1.5.7), with the co‐crystallized ligand pose serving as a reference. The grid parameters were chosen to be sufficiently wide so as not to force the ligand‐receptor interaction. The grid parameters were center *x* = 17.324, center *y* = 40.930, center *z* = 9.178, npts *x* = 22, npts *y* = 30, npts *z* = 26, and spacing = 1. The grid parameters adapted for post‐MD redocking of all cluster representatives are listed in Table S2. All data concerning the molecular docking analyses performed in the various stages described in this manuscript, three‐dimensional structures of proteins and ligands in input and output, are available in the public GitHub repository at https://github.com/rocco‐b/SYK‐Kinase‐Inhibitors‐Docking‐and‐MD‐data.

### 2.4 Molecular Dynamics Simulations

MD simulations were performed using the YASARA software. The cubic simulation cell was extended for 5 Å around all protein atoms, applying periodic boundary conditions in all directions, and MD was performed using the md_runfast integrated macro. The simulation was performed at physiological pH in a physiological water solution (0.9% NaCl) at 298 K, and the water density was 0.997 g/mL. For the Van der Waals forces, the cut‐off was 8 Å, and no cut‐off was applied to the electrostatic forces (using the Long‐range Coulomb algorithm). MD simulations were performed for 200 ns using the ff14SB force field. After the MD simulation, the RMSD, RMSF, internal energy, SASA, and PCA trends over time were evaluated using the md_analyze macro. MD trajectory cluster analysis was also performed using the md_analyze macro, with an RMSD threshold of 2.5 Å calculated for the heavy atoms. Clustering was performed on the equilibrated portions of the trajectories. MM/PBSA analysis was performed using the md_analyzebindenergy macro. The use of YASARA's built‐in macros guarantees standardized parameters, ensuring high reproducibility of the computational pipeline. According to the YASARA energy calculation convention, binding energy was calculated as in Equation (1). Therefore, positive values indicate thermodynamically favorable binding.

(1) $$\begin{array}{c} \text{BindEnergy}=E_{\mathrm{pot}}\;\text{Recept}+E_{\text{solv}}\;\text{Recept}+E_{\mathrm{pot}}\;\text{Ligand}\\ \;+E_{\text{solv}}\;\text{Ligand}-E_{\mathrm{pot}}\;\text{Complex}-E_{\text{solv}}\;\text{Complex} \end{array}$$

### 2.5 SAveRUNNER Analysis

SAveRUNNER (Fiscon and Paci 2021) is an R‐based software and was run as described in the User Guide available on its GitHub repository. The input files were updated compared with the original version. Specifically, AT‐associated genes were updated with information retrieved from the U.S. Centers for Disease Control and Prevention (CDC, https://www.cdc.gov/), and drug‐target interactions were updated with information retrieved from the Drug‐Gene Interaction Database (DGIdb, https://dgidb.org) (Cannon et al. 2024).

### 2.6 RMSD Calculation

RMSD calculations were performed using the obrms command in Open Babel (v. 3.1.1) (O'Boyle et al. 2011). The co‐crystallized ligand and docking output poses were provided in PDB format.

### 2.7 Ligand‐Protein Interactions Analysis

Ligand‐protein interactions were analyzed using BIOVIA Discovery Studio Visualizer (v. 25.1.0.24284, Dassault Systèmes Biovia Corp., San Diego, CA, USA). The co‐crystallized structure was uploaded in PDB format, and all hydrogen atoms were added using the “Chemistry → Hydrogen → Add” option in the upper toolbar. The co‐crystallized pose was selected and defined as the ligand. The interactions were analyzed using the “Show 2D Diagram” option.

### 2.8 Free Energy Landscape (FEL) Calculations

The FEL of each ligand‐receptor complex was reconstructed from the PCA trajectories using a custom Python script (Papaleo et al. 2009). The two‐dimensional probability density distribution, *P*(*x*, *y*), of the conformational states sampled along the first two principal components (PC1 and PC2) was estimated via Gaussian Kernel Density Estimation (KDE) using the SciPy library. The probability density was subsequently converted into free energy (Δ*G*) by applying the inverse Boltzmann Equation (2):

(2) $$\Delta G=-k_{B}T\ln P\left(x,y\right)$$

where *k*_B_ is the Boltzmann constant, and *T* is the simulation temperature (298 K). The global energy minimum of each landscape was normalized to 0 kcal/mol as the baseline, and a maximum energy cutoff of 6.0 kcal/mol was applied to enhance the visualization of the biologically relevant low‐energy basins. High‐resolution contour maps and three‐dimensional surface plots were generated using the Matplotlib library. To ensure full computational reproducibility and methodological transparency, the complete Python code used for FEL generation is freely accessible in a dedicated GitHub repository (https://github.com/rocco‐b/SYK‐Kinase‐Inhibitors‐Docking‐and‐MD‐data).

## 3 Results and Discussion

### 3.1 Structural Rationale and Target Characterization

SYK is a cytoplasmic non‐receptor tyrosine kinase that is abundantly expressed in microglial cells and multiple immune cell populations, including monocytes, macrophages, and natural killer (NK) cells. This protein exhibits a highly conserved modular architecture that underlies its tightly regulated activation mechanism. Structurally, SYK comprises two Src homology 2 (SH2) domains at the *N*‐terminus, *N*‐SH2 (residues 14–106) and C‐SH2 (residues 167–258), followed by a *C*‐terminal kinase domain (residues 364–620), with these functional modules separated by interdomain regions A and B, the latter spanning residues 259–363 (Mócsai et al. 2010). In resting immune cells, tandem SH2 domains adopt an autoinhibitory “linker–kinase sandwich” conformation that stabilizes SYK in an inactive state by restricting access to the catalytic domain. Upon immune receptor engagement, the SH2 domains bind to phosphorylated tyrosine residues within immunoreceptor tyrosine‐based activation motifs (ITAMs), triggering a conformational rearrangement that releases the kinase domain and initiates SYK activation. This process enables the phosphorylation of key tyrosine residues within interdomains A and B, as well as within the C‐terminal tail, through auto‐phosphorylation and/or trans‐phosphorylation mediated by the Src‐family kinase Lyn (Pamuk and Tsokos 2010). These phosphorylation events are essential for full catalytic activation and subsequent recruitment of downstream substrates.

In AT, aberrant activation of SYK downstream of the adaptor protein TYROBP sustains a pro‐inflammatory signaling state, thereby exacerbating microglial activation, neuronal loss, and progressive neurological impairment. Given the central role of the TYROBP–SYK axis in amplifying neuroinflammatory cascades, pharmacological inhibition of SYK represents a rational strategy for attenuating inflammation‐driven neurodegeneration. Notably, TYROBP has emerged as a therapeutic target in other neurodegenerative disorders, including Alzheimer's disease and amyotrophic lateral sclerosis (ALS), further supporting the translational relevance of this pathway in AT (Haure‐Mirande et al. 2022). Accordingly, the present study focuses on targeting the ATP‐binding pocket of the SYK kinase domain to disrupt pathogenic signaling at the molecular core.

### 3.2 Protein Model Validation

The SYK protein structure (PDB ID: 3EMG) was retrieved from the PDB‐Redo repository (https://pdb‐redo.eu/) because this refined model exhibited improved overall structural quality parameters compared to the original co‐crystal structure deposited in the Protein Data Bank (https://www.rcsb.org/). The selected model was resolved at a resolution of 2.60 Å, and the co‐crystallized ligand was associated with an experimentally determined inhibition constant (*K*_i_) of 9 nM.

Before molecular docking, the co‐crystallized ligand was removed from the ATP‐binding site, and redocking was performed using the GNINA docking algorithm. GNINA enhances conventional docking approaches by integrating convolutional neural networks (CNNs) into the scoring functions, thereby improving pose prediction and affinity estimation. The ligand conformational space was explored using a Monte Carlo (MC) sampling strategy, which applies random translations, rotations, and torsional modifications to the ligand. Each MC step was followed by local energy minimization, and the best‐scoring conformations were retained for further evaluation.

Depending on user‐defined settings, CNN‐based scoring can be applied at different stages of docking workflows. In the present study, GNINA was operated in rescoring mode, in which a predefined CNN model was used to evaluate and rank the generated ligand poses based on their predicted binding affinities. Two primary metrics were employed to assess ligand–protein interactions: (i) CNN affinity, which estimates the dissociation constant in terms of the predicted p*K*_d_, and (ii) CNN score, an internal GNINA metric reflecting the structural quality of the predicted binding pose. The product of these two values, referred to as CNN_VS, has been recommended by GNINA developers as a more reliable predictor of ligand‐binding affinity. The predicted p*K*_d_ values were subsequently converted into dissociation constants (*K*_d_), expressed in nanomolar units, using Equation (3):

(3) $$K_{d}=10^{\left(9-pKd\right)}$$

**FIGURE 1:**
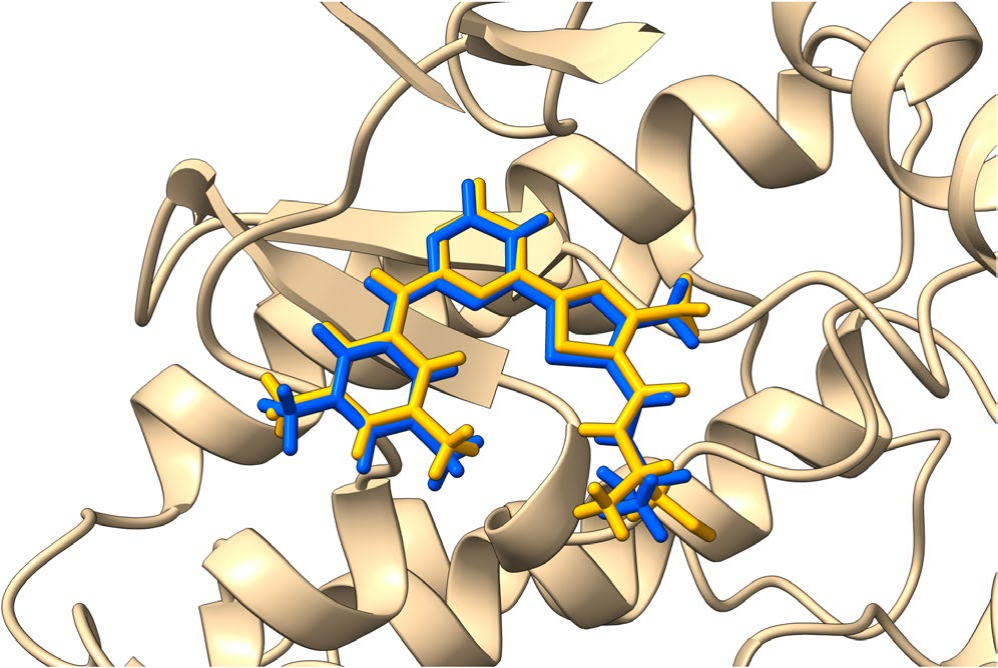
Comparison between the co‐crystallized ligand pose (orange) and the reproduced pose using GNINA (blue).

The protein model was validated using a rigorous protocol established in our previous studies (Buccheri and Rescifina 2025), which included redocking of the co‐crystallized ligand and statistical validation against a decoy set. The docking procedure successfully reproduced the experimental binding pose of the co‐crystallized ligand (Figure 1), yielding a root mean square deviation (RMSD) of 1.00 Å (Ramírez and Caballero 2018). The CNN score for the reproduced pose was 0.99, indicating an excellent pose quality. Moreover, the calculated CNN_VS value was 7.77, corresponding to a predicted *K*_d_ of 16.98 nM, which is in close agreement with the experimentally determined *K*_i_ value. These results confirm the ability of GNINA to accurately reproduce the native binding mode and provide a reliable estimate of the ligand–protein binding affinity.

To further assess the discriminatory power of the docking protocol, statistical validation was conducted to evaluate its capability to distinguish between active ligands and theoretically inactive decoy molecules. The results of this analysis, previously reported by our group (Buccheri and Rescifina 2025), yielded a receiver operating characteristic area under the curve (ROC‐AUC) of 0.90, with enrichment factors of 10, 12, and 7 in the top 1%, 5%, and 10% of the ranked database (EF1, EF5, and EF10), respectively.

These performance metrics substantially exceeded those expected under random selection (AUC = 0.50; EF = 1.0), demonstrating that the adopted docking protocol yielded a substantially higher hit rate. Collectively, the RMSD, predicted binding affinity, ROC‐AUC, and enrichment factor values indicated that the GNINA‐based docking workflow was robust, reliable, and highly effective in discriminating active from inactive compounds. This validation confirmed the suitability of the selected SYK protein model for subsequent virtual screening.

### 3.3 Virtual Screening

Following the validation of the docking protocol, virtual screening (VS) was performed on a curated library of 2342 FDA‐approved drugs. The docking results were evaluated using the CNN_VS scoring function, and a threshold value of 6.30 was applied. As CNN_VS predicts ligand–protein interaction strength in terms of p*K*_d_, this cutoff corresponds to an estimated dissociation constant (*K*_d_) of 500 nM, a value widely considered to be indicative of biologically relevant kinase inhibition (Mysinger et al. 2012). Accordingly, only compounds with CNN_VS ≥ 6.30 (i.e., *K*_d_ ≤ 500 nM) were retained for further analysis. This filtering step yielded 43 compounds, which were subsequently subjected to detailed visual inspection and interaction analysis within the SYK ATP‐binding site. Structural evaluation was guided by the interaction patterns commonly observed in experimentally validated SYK inhibitors. In particular, inhibitor efficacy is strongly associated with the ability to engage key residues within the hinge region and DFG motif, both of which are critical for kinase catalytic activity.

The hinge region, located within the kinase domain, is essential for ATP recognition and alignment and serves as the primary anchoring site for small‐molecule inhibitors via hydrogen‐bonding interactions. The DFG (Asp‐Phe‐Gly) motif is a highly conserved structural element in tyrosine kinases that regulates catalytic activity by controlling the conformational transitions between the active and inactive states (Ung and Schlessinger 2015). Consequently, the following interactions were considered essential for effective SYK inhibition: (i) hydrogen bonding with Ala451 (Singh et al. 2012; Huang et al. 2017; Marchetti et al. 2020; Mansouri et al. 2024); (ii) hydrophobic or van der Waals interactions with Met448 (Huang et al. 2017), Met450 (Marchetti et al. 2020), and Pro455 (Singh et al. 2012; Huang et al. 2017; Marchetti et al. 2020; Mansouri et al. 2024) within the hinge region; and (iii) hydrogen‐bond or electrostatic interaction with Asp512 of the DFG motif (Singh et al. 2012; Huang et al. 2017; Marchetti et al. 2020; Mansouri et al. 2024).

The presence or absence of these interactions for each compound is summarized in Table 1, where a value of 1 indicates the occurrence of the corresponding ligand–residue interaction, and 0 denotes its absence. Compounds exhibiting all five interactions were considered optimal candidates for SYK inhibition (Table 1).

**TABLE 1:** Intermolecular interactions identified for the selected ligands during VS.

ID	CNN_VS	Ala451	Met450	Pro455	Met448	Asp512
880	6.34	0	1	0	1	1
932	6.54	1	1	1	1	1
973	6.89	1	1	0	1	0
999	6.45	0	1	0	1	1
1017	6.69	1	1	0	0	0
1088	6.63	1	1	1	1	0
1098	6.57	1	1	1	1	0
1109	6.75	0	1	0	1	1
1130	6.40	1	1	0	1	1
1142	6.42	1	1	1	1	0
1260	6.56	0	1	0	1	1
1278	6.49	1	1	1	1	0
1304	6.39	0	1	0	1	0
1305	6.59	1	1	1	1	0
1365	7.03	1	1	1	0	0
1425	6.49	1	1	1	0	1
1496	6.42	1	1	0	1	0
1537	6.37	1	1	1	1	0
1541	6.71	1	1	0	1	1
1547	6.39	1	1	0	0	0
1561	6.39	0	1	1	1	0
1595	6.71	1	1	1	1	0
1639	6.65	0	1	0	1	0
1650	7.06	0	1	1	1	1
1655	7.31	1	1	1	0	0
1699	6.31	0	1	1	1	0
1701	6.75	1	1	0	0	1
1705	6.84	0	0	0	1	0
1706	7.49	1	1	1	1	1
1714	7.65	1	1	1	1	0
1836	6.65	1	1	1	1	1
1855	7.41	1	1	1	1	0
1863	7.56	1	1	1	1	1
1872	6.83	1	1	0	0	1
1912	7.30	1	1	1	1	0
2061	8.00	1	1	1	1	1
2066	6.66	1	1	0	1	0
2104	7.21	1	1	1	1	0
2113	6.48	1	1	1	1	1
2119	7.18	1	1	1	1	0
2147	7.48	1	1	1	1	1
2180	6.73	1	0	0	1	1
2218	6.66	1	1	1	1	1

*Note:* A value of 1 indicates the presence of the corresponding ligand–residue interaction, whereas 0 indicates its absence. The highlighted rows correspond to the ligands exhibiting all interactions of interest.

Analysis of the interaction profiles revealed that eight FDA‐approved compounds satisfied all the structural criteria. The compounds olanzapine (ID: 932), momelotinib (ID: 1706), vemurafenib (ID: 1836), palbociclib (ID: 1863), imatinib (ID: 2061), nilotinib (ID: 2113), flumatinib (ID: 2147), and riboflavin (ID: 2218) were identified as the most promising candidates for repositioning as SYK kinase inhibitors.

### 3.4 SAveRUNNER Genomic Analysis

Given that the primary objective of this study was drug repositioning for AT, the Searching off‐lAbel dRUg aNd NEtwOrk (SAveRUNNER) platform was employed to integrate structure‐based predictions with network‐level biological relevance. SAveRUNNER is a freely available computational tool designed to identify novel therapeutic indications for drugs that are already approved or marketed, based on principles derived from network medicine. The theoretical framework of SAveRUNNER is grounded in the concept that diseases arise from localized perturbations within the human protein–protein interaction network (interactome). Disease‐associated genes tend to cluster within specific regions of the interactome, forming disease modules. Similarly, drug action can be interpreted as perturbing the same network, and both therapeutic efficacy and adverse effects depend on the topological proximity between drug targets and disease modules. SAveRUNNER predicts drug–disease associations by quantifying the network‐based proximity between drug targets and disease‐associated proteins using an adjusted similarity measure that prioritizes associations localized within the same interactome regions (Fiscon and Paci 2021).

**FIGURE 2:**
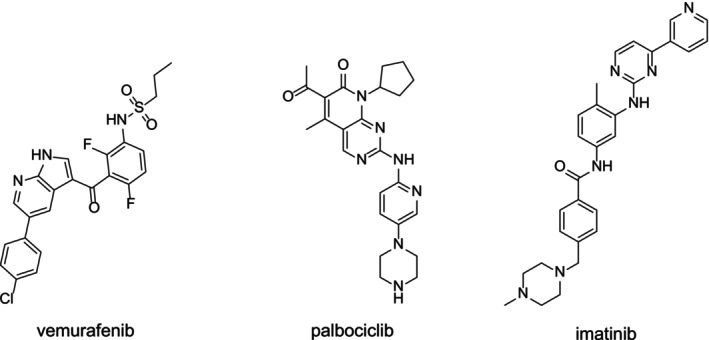
Two‐dimensional chemical structures of selected drug candidates proposed for repositioning as SYK kinase inhibitors.

The SAveRUNNER output (Table S1) was analyzed by applying a stringent adjusted similarity threshold (> 0.96), which was considered indicative of a high‐confidence candidate for repositioning. To strengthen the robustness of candidate selection, a cross‐validation step was performed to verify whether the compounds identified by structure‐based virtual screening (GNINA) were predicted to be potentially active against AT by SAveRUNNER. This orthogonal filtering strategy identified three compounds (Figure 2) that met both criteria and were selected for subsequent molecular dynamics (MD) simulations.

The remaining five compounds initially identified during the virtual screening phase (olanzapine, momelotinib, nilotinib, flumatinib, and riboflavin) were excluded because they failed to meet the adjusted similarity threshold in the SAveRUNNER analysis. Although these molecules exhibited favorable structural compatibility with the SYK ATP‐binding site, their predicted influence on the AT disease module within the human interactome was comparatively weak. This discrepancy highlights the importance of integrating network‐level information with structure‐based affinity predictions, as a high binding affinity alone does not necessarily translate into disease‐modifying potential.

By requiring candidate compounds to pass both the GNINA‐based structure‐driven filter and the SAveRUNNER‐based network‐driven filter, we implemented an orthogonal validation strategy that combined molecular fitting with biological relevance. This approach ensured that the final lead compounds (vemurafenib, palbociclib, and imatinib) exhibited stable and favorable interactions with SYK at the molecular level and a statistically significant topological association with the AT disease module, thereby strengthening their potential for effective drug repositioning.

The selected drugs included vemurafenib (ID: 1836), palbociclib (ID: 1863), and imatinib (ID: 2061). Vemurafenib is a selective inhibitor of BRAF kinase and has been approved by the FDA for the treatment of patients with unresectable or metastatic melanoma harboring the BRAFV600E mutation (Flaherty et al. 2011). Palbociclib is a cyclin‐dependent kinase (CDK) 4/6 inhibitor that was developed for the treatment of hormone receptor–positive breast cancer (Dhillon 2015). Imatinib is a tyrosine kinase inhibitor with activity against BCR–ABL, which was originally developed for the treatment of chronic myeloid leukemia (CML) (Lyseng‐Williamson and Jarvis 2001).

### 3.5 Homology Modeling

Because the available co‐crystallized SYK structure was incomplete and a full‐length protein model was required to accurately capture long‐range conformational dynamics during MD simulations, a homology modeling approach was employed. The inclusion of the missing regions was considered essential, as large‐scale domain motions and interdomain flexibility can indirectly modulate ligand–protein interactions within the ATP‐binding site over extended simulation timescales. The complete human SYK amino acid sequence was retrieved from UniProt (UniProt ID: P43405; https://www.uniprot.org/) and used as input for homology modeling in the FASTA format. The experimentally resolved co‐crystallized structure was adopted as the primary template to preserve the native conformation of the kinase domain and ligand‐binding region. Missing segments were reconstructed based on sequence homology and structurally compatible regions, yielding a hybrid, full‐length SYK model.

**FIGURE 3:**
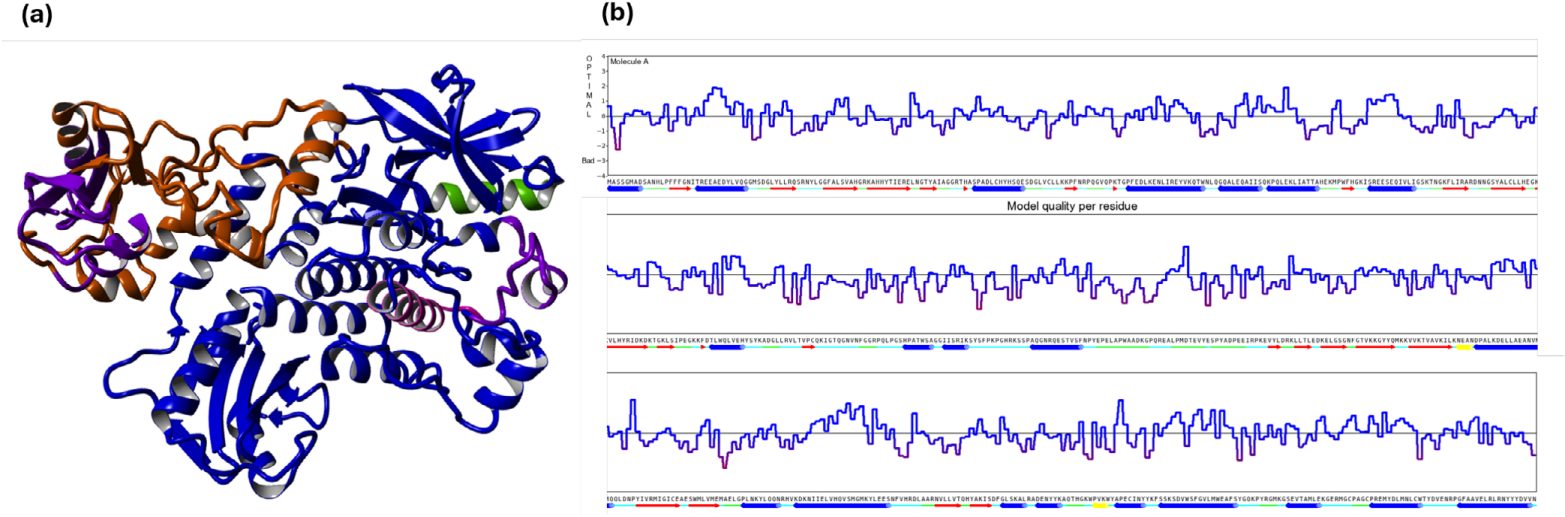
(a) Homology model of full‐length SYK. The experimentally resolved regions derived from the initial co‐crystallized structure are shown in blue, whereas the reconstructed (hybridized) regions obtained by homology modeling are highlighted in distinct colors. (b) The graph illustrates the local quality expressed as Z‐scores (standard deviations from the average high‐resolution X‐ray structures). Positive scores denote optimal conformations, whereas negative scores indicate regions with suboptimal packing or dihedral angles. An overall Z‐score of −0.547 indicates the global correctness of the globular structure.

The resulting homology model (Figure 3) retained the experimentally determined structural core while incorporating previously unresolved regions required for dynamic simulations. The hybridization and structural validation of the missing loops were handled natively by YASARA's automated homology modeling module, which intrinsically applies energy minimization to ensure stereochemical quality. This complete model was subsequently used as the initial structure for all MD simulations.

### 3.6 Molecular Dynamics Simulations

**FIGURE 4:**
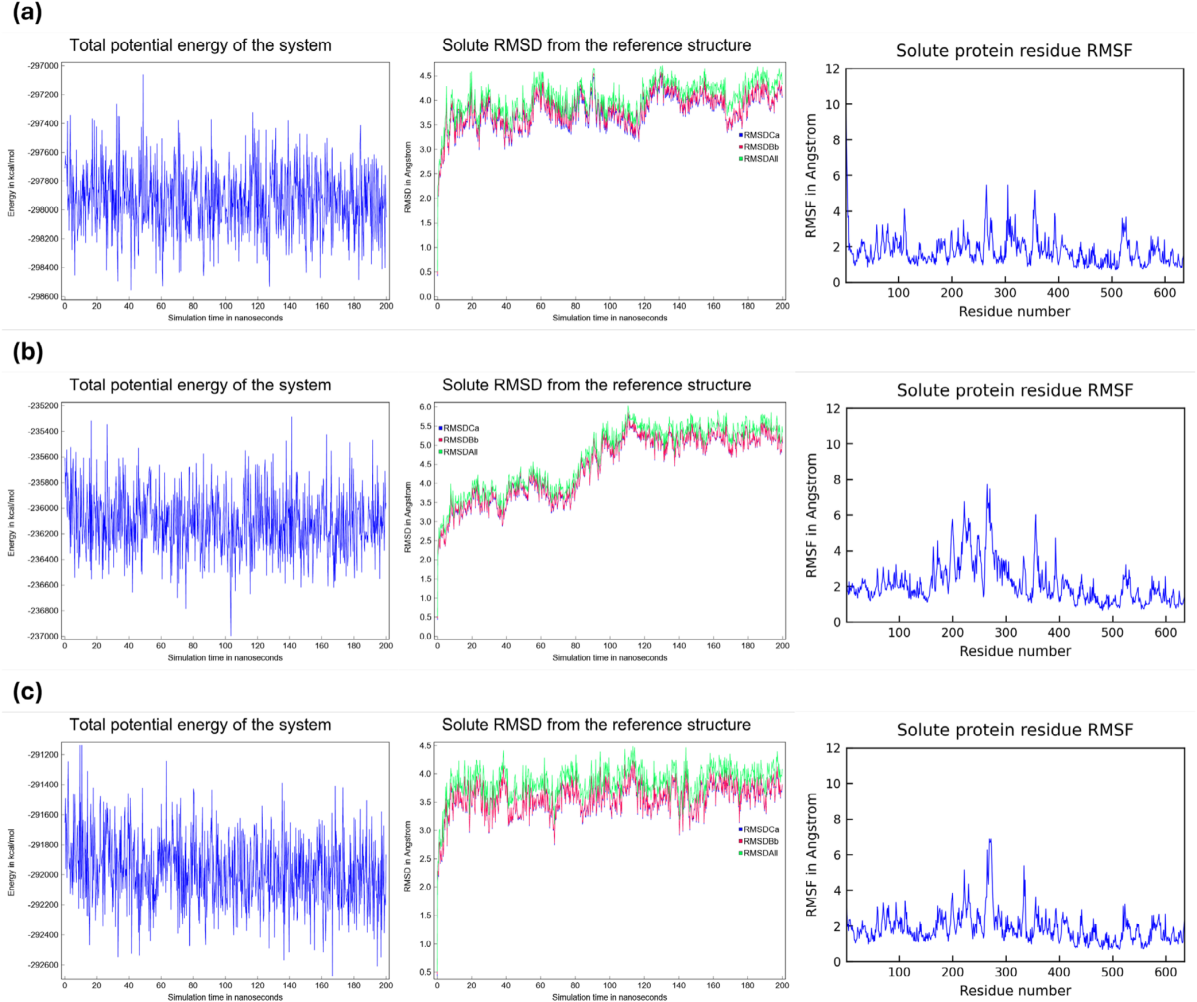
Left: Total potential energy of the system as a function of time (200 ns) for the three ligand–protein complexes. Center: solute RMSD from the reference structure as a function of time (200 ns). The plot shows the alpha carbons (RMSDCa, blue), backbone (RMSDBb, red), and all‐heavy atom (RMSDAll, green) RMSDs. Right: Root‐Mean‐Square Fluctuation (RMSF) per solute protein residue calculated from the average RMSF of the atoms constituting the residue. Panels: (a) vemurafenib, (b) palbociclib, and (c) imatinib.

The docked binding poses of the selected ligands were first aligned to the full‐length homology model of SYK and subsequently subjected to energy minimization to relax local steric clashes and obtain energetically favorable starting conformations of the ligand–protein complexes for MD simulations. All‐atom MD simulations were then performed for each of the three selected drug candidates over a timescale of 200 ns, with the aim of assessing the conformational stability of the complexes under cytoplasm‐like conditions. During each simulation, the total potential energy of the system and the root‐mean‐square deviation (RMSD) of the solute relative to the reference structure were monitored as the primary indicators of system equilibration and structural stability (Figure 4). The initial energy‐minimized full‐length homology model was used as the reference structure for all RMSD calculations.

As shown in the left panels of Figure 4, the potential energy profiles of all three systems fluctuated around stable mean values throughout the production phase, with no evidence of systematic drift, indicating that the simulations reached a thermodynamically stable regime. The solute RMSD trajectories (Figure 4, center panels) exhibited an initial increase during the early stages of the simulations, corresponding to structural relaxation from the starting configurations, followed by plateau‐like fluctuations for the remainder of the trajectories.

The onset of the apparent equilibrium regime occurred at different times for each of the complexes. Specifically, RMSD stabilization was observed after approximately 10 ns for the vemurafenib–SYK and imatinib–SYK complexes, whereas the palbociclib–SYK system required a longer equilibration period, reaching a stable RMSD plateau after approximately 110 ns of simulation. Beyond these time points, the RMSD values fluctuated around the system‐specific mean values for the remainder of the 200 ns simulations.

Collectively, the absence of energy drift and the convergence of the RMSD profiles indicated that all ligand–protein complexes sampled stable conformational ensembles over the majority of the simulation time, supporting the suitability of the generated trajectories for subsequent structural and interaction analyses.

Root‐mean‐square fluctuation (RMSF) analysis was conducted to evaluate residue‐level flexibility across the simulation trajectories. As expected, the residues in the binding site that directly interacted with the ligands exhibited significantly reduced fluctuations (Figure 4, right panel), particularly in the 450–520 region, where the residues critical for protein‐ligand interaction were located. Specifically, this stabilized segment encompasses the key structural elements of the SYK catalytic domain, including the hinge region (comprising Met448, Met450, Ala451, and Pro455) and the highly conserved DFG motif (Asp512). The restricted mobility of these specific amino acids throughout the 200 ns simulations strongly suggested that the ligands acted as conformational locks, maintaining persistent and rigid structural anchorage within the ATP‐binding pocket.

Following the assessment of global complex stability and local residue flexibility, a detailed temporal analysis of the intermolecular interactions was performed to validate our mechanistic hypothesis. In addition, hydrogen bond and hydrophobic contact occupancies were monitored throughout the 200 ns trajectory (Figure S1). Persistent interactions (> 50% occupancy) were observed for the key residues previously identified in the docking analysis, specifically encompassing the hinge region (Met448, Met450, Ala451, and Pro455) and the DFG motif (Asp512). The maintenance of these critical contacts over the simulation timescale confirmed that the ligands did not undergo significant orientational shifts after protein relaxation, fully supporting the structural relevance and dynamic stability of the predicted binding modes under explicit solvent conditions.

To further evaluate the global structural integrity and compactness of the SYK kinase domain during the simulations, the Solvent Accessible Surface Area (SASA) was calculated (Figure S2). The mean per‐residue SASA profiles (Figure S2, left panels) demonstrated highly consistent solvation patterns across all three complexes, confirming that the overall globular fold of the protein was strictly preserved. Furthermore, the time‐evolution heatmaps of the per‐residue SASA (Figure S2, right panels) exhibited continuous, stable horizontal bands over the entire 200 ns trajectories, indicating steady solvation states for both buried and surface‐exposed residues. This global structural conservation is of critical importance, as it demonstrates that the marked instability of the imatinib complex, previously detected via RMSD and binding energy fluctuations, is driven by localized ligand unbinding and binding‐pocket breathing, rather than a macroscopic denaturation of the receptor. Conversely, the stable SASA profiles for vemurafenib and palbociclib provide supplementary evidence of a persistently packed, stable ligand‐receptor interface that excludes the solvent effectively throughout the dynamic relaxation.

### 3.7 Principal Component Analysis and Free Energy Landscape

To delineate the essential large‐scale motions of the SYK kinase domain upon ligand binding, PCA was performed independently on the trajectory of each complex. The PCA eigenvalue curve (Figure S3, left panels) demonstrated an asymptotic decay for all systems, confirming that the first two principal components (PC1 and PC2) account for most of the total concerted motions, thus validating their use as reaction coordinates for further thermodynamic analyses.

The projection of the trajectories onto PC1 and PC2 over the 200 ns simulation time (Figure S3, central and right panels) provided a direct readout of structural convergence. The palbociclib complex exhibited exceptional conformational locking; after an initial structural relaxation phase, its primary motion axis (PC1) settled into a perfectly flat plateau from ~100 ns onwards, resulting in a highly dense and localized cluster in the 2D scatter space. This rigid behavior aligns with its low RMSD and robust binding affinity. The vemurafenib complex also demonstrated overall stability, exploring a confined region of the conformational space characterized by discrete, localized sub‐states rather than continuous structural drift. Conversely, the PCA trajectory for imatinib corroborated its marked instability. Both PC1 and PC2 failed to achieve a stable plateau, exhibiting continuous and chaotic drift throughout the entire 200 ns simulation. The corresponding 2D projection revealed a highly diffuse scatter pattern lacking a defined core cluster, confirming that the imatinib‐SYK complex explores a vast, unstable conformational space without reaching a thermodynamically stable binding mode.

**FIGURE 5:**
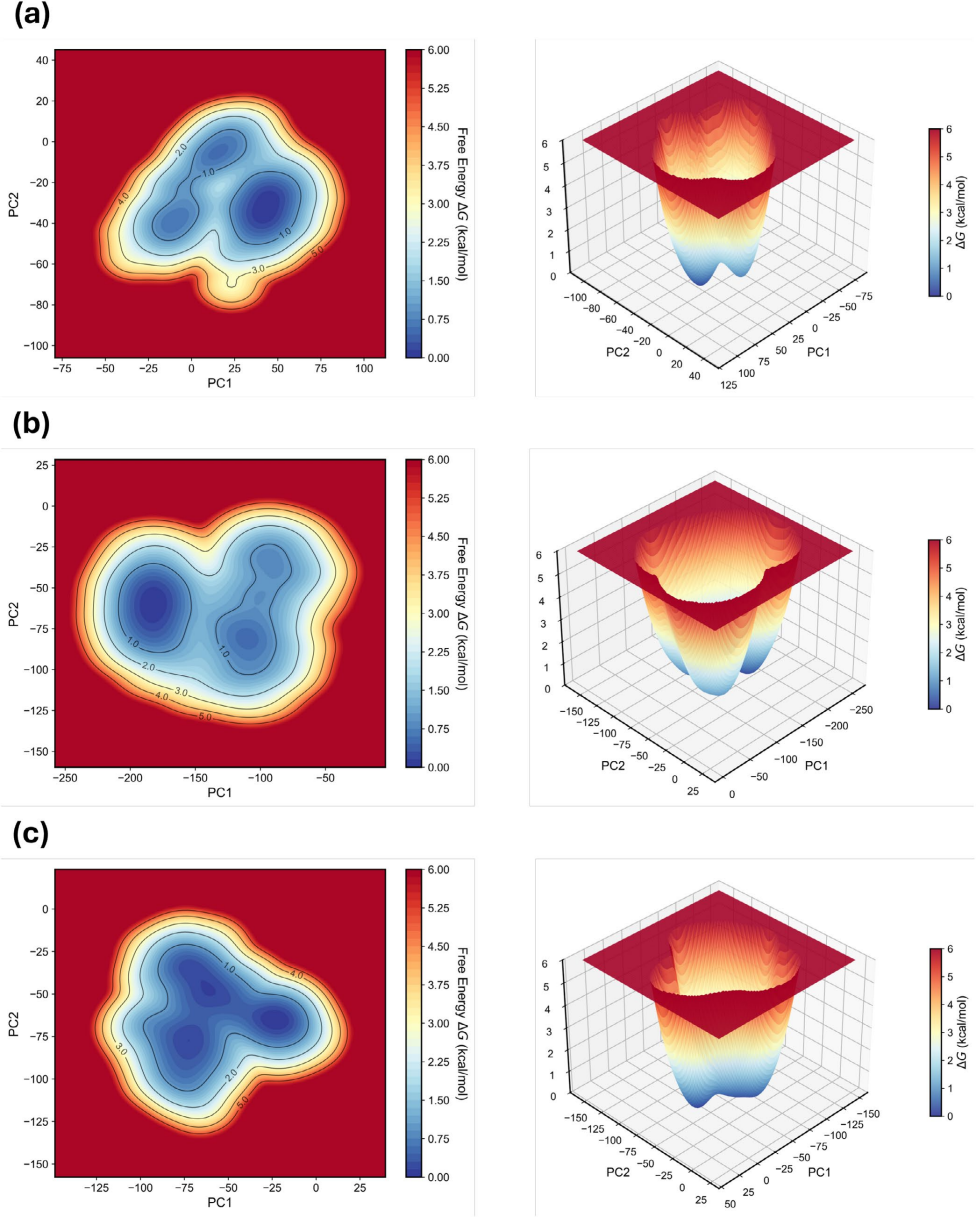
FEL of the SYK kinase complexed with repurposed drug candidates. Left: Two‐dimensional contour map. Right: Three‐dimensional surface plots. Panels: (a) vemurafenib, (b) palbociclib, and (c) imatinib.

To further characterize the thermodynamic stability of the ligand‐receptor complexes and map their conformational space, FELs were derived from the PCA projections of the MD trajectories (Figure 5). Because the PCA was performed independently for each complex to accurately capture ligand‐specific induced dynamics, the principal components represent trajectory‐specific eigenvectors. Therefore, our comparative analysis focused on the topological and thermodynamic features of the landscapes, such as the broadness of the basins and the depth of the energetic minima, rather than their absolute spatial coordinates.

The FEL of the vemurafenib complex (Figure 5a) displayed a confined and well‐defined low‐energy basin. This topology indicates that the complex preferentially samples a limited conformational region, with reduced structural dispersion, consistent with the low RMSF values observed in the active‐site region. Overall, these data support a stabilizing role of vemurafenib on the SYK catalytic pocket rather than suggesting broad conformational rearrangements during the simulation.

Similarly, palbociclib (Figure 5b) exhibited a well‐defined energetic basin with deep minima. Although slightly broader than vemurafenib, suggesting a localized flexibility necessary to accommodate the ligand, the landscape remained bounded by steep energy walls, indicating a robust and persistent interaction without unfolding or drifting.

In stark contrast, the FEL of imatinib (Figure 5c) reflected highly perturbed conformational behavior. Although a minimum is visually identifiable, the underlying dynamics correlate with the critically high RMSD (10.53 Å), and the significant loss of binding affinity observed in MM/PBSA calculations (Section 3.8). The independent PCA for imatinib captured macroscopic structural deviations and complex destabilization, highlighting the inability of this drug to maintain a stable anchoring within the MD‐refined SYK catalytic pocket. Collectively, these thermodynamic landscapes provide a strong structural rationale supporting vemurafenib and palbociclib as the most viable repurposed candidates.

### 3.8 Redocking Analysis and MM/PBSA Calculations

Redocking involves repeating the docking procedure on protein conformations obtained after structural refinement, such as molecular dynamics (MD) simulations. This strategy enables the validation of ligand–protein complex stability and refinement of binding poses within an energetically relaxed and dynamically relevant receptor conformation, thereby partially accounting for induced‐fit effects (Pinzi and Rastelli 2019; Lexa and Carlson 2012; Salmaso and Moro 2018). In this study, an ensemble‐based post‐MD redocking protocol was employed to reassess the initially predicted binding affinities in the context of protein conformations shaped by long timescale dynamics. The MD trajectories were clustered from the equilibration point onwards (after 10 ns for vemurafenib and imatinib, and after 110 ns for palbociclib) based on the structural similarity assessed in terms of RMSD (De Paris et al. 2015). Trajectory clustering yielded a representative set of dynamically relaxed snapshots for each system: 10 for vemurafenib, 3 for palbociclib, and 11 for imatinib complexes.

For each extracted snapshot, a cubic grid was constructed around the ligand (Table S2). The ligand was subsequently removed and redocked using the same parameters as those applied during the initial virtual screening phase. To evaluate the results, both the CNN_VS score and RMSD of the redocked pose relative to the corresponding MD conformation were calculated. Furthermore, to support the redocking results with comprehensive thermodynamic data, MM/PBSA analysis was performed over the entire 200 ns MD trajectory for each complex. The solvation energies were calculated for each snapshot using the Poisson‐Boltzmann (PBS) method in YASARA, allowing us to monitor the binding energy trends over time.

The results of the redocking analysis (Tables S3–S5) confirmed the binding affinity and conformational stability of vemurafenib and palbociclib. Both compounds exhibited high average CNN_VS scores and remarkably low RMSD values (Table 2). These low RMSD values indicate that the redocked poses were highly conserved and perfectly compatible with the dynamically relaxed receptor conformations. Moreover, the time‐dependent MM/PBSA energy profiles (Figure S4) spanning the 200 ns simulations revealed that vemurafenib and palbociclib formed energetically stable ligand‐protein complexes, consistent with the redocking results. Specifically, they exhibited remarkably high and comparable thermodynamic affinities, with average binding energies of 53.583 kcal/mol and 52.448 kcal/mol, respectively (Table S6).

**TABLE 2:** Average redocking performance calculated across all cluster representatives extracted from the MD trajectories.

Compound	Number of clusters	CNN_VS	RMSD (Å)
Vemurafenib	10	7.60 ± 0.21	1.34 ± 0.44
Palbociclib	3	7.69 ± 0.22	1.50 ± 0.51
Imatinib	11	2.16 ± 1.47	10.53 ± 1.66

*Note:* Data are expressed as mean ± standard deviation for both the predicted affinity (CNN_VS) and positional deviation (RMSD) of the redocked poses relative to their corresponding MD conformations.

In contrast, a pronounced loss of affinity was observed with imatinib. The compound exhibited a substantial reduction in the average CNN_VS score and a critically high average RMSD, demonstrating a complete inability to achieve a stable binding pose within the MD‐refined receptor conformation. Consistent with these structural and scoring metrics, the MM/PBSA analysis showed a remarkably less favorable energetic profile for the imatinib complex over the simulated timeframe compared with the other two drugs, yielding a significantly lower average binding energy of 15.218 kcal/mol (Table S6). This quantitative drop (more than 3.4‐fold lower than that of vemurafenib and palbociclib) confirms a marked decrease in ligand‐protein interaction stability, despite the favorable initial static docking results.

From a translational perspective, an essential requirement for the therapeutic relevance of AT is the ability of candidate compounds to cross the blood–brain barrier (BBB) and access the central nervous system. Vemurafenib and palbociclib are known substrates of efflux transporters, such as P‐glycoprotein (P‐gp) and breast cancer resistance protein (BCRP), which limit baseline CNS penetration. Nevertheless, accumulating clinical and preclinical evidence indicates that both compounds reach pharmacologically relevant concentrations in the brain. Vemurafenib has demonstrated significant clinical efficacy in patients with BRAF‐mutant melanoma brain metastases, providing direct evidence of intracranial target engagement (McArthur et al. 2017). Similarly, palbociclib has shown measurable intracranial antitumor activity in selected brain tumor models, supporting its capacity to access CNS targets (Raub et al. 2015).

Importantly, in the pathological context of AT, chronic neuroinflammation is frequently associated with localized disruption of BBB integrity, resulting in a “leaky” barrier that may further facilitate drug penetration into the affected cerebellar regions (Luo and Qiao 2026). Collectively, these findings support the prioritization of vemurafenib and palbociclib for further preclinical evaluation in AT, with a plausible therapeutic window for modulating microglial SYK signaling in vivo.

### 3.9 Comparison With Representative SYK Inhibitors

To place the ensemble docking results in a validated pharmacological context, the top‐ranked repurposed candidates were directly compared with the known SYK inhibitors previously evaluated using the same GNINA workflow, receptor model, and docking protocol (Table 3) (Buccheri and Rescifina 2025). In the benchmark dataset, which included 80 literature SYK ligands with experimental affinity values, the top‐performing inhibitors exhibited CNN_VS values of up to 7.91, whereas the co‐crystallized reference ligand yielded a CNN_VS value of 7.85. The full dataset, comprising all 80 previously reported inhibitors and their corresponding values, is freely available in the associated GitHub repository (https://github.com/rocco‐b/HTHQ‐GNINA). It should be emphasized that the CNN_VS values reported for the known inhibitors and the co‐crystallized ligand are single‐structure docking scores drawn from the benchmark dataset, whereas those of the repurposed candidates are mean values obtained from post‐MD ensemble redocking. Accordingly, this comparison is intended to position the repurposed hits within the affinity range spanned by the experimentally validated SYK inhibitors under a common GNINA scoring scheme, rather than to constitute a strict head‐to‐head ranking under identical conformational sampling.

**TABLE 3:** Comparison of repurposed candidates and known SYK inhibitors evaluated using the same GNINA workflow.^a^

Compound	Type	Experimental *K*_i_ (nM)	CNN_VS (benchmark or ensemble)	RMSD (Å)
Co‐crystallized ligand	Reference ligand	9	7.85	0.97
Demethyl imatinib	Known inhibitor	160	7.91	—
R343	Known inhibitor	1.8	7.67	—
Vemurafenib	Repurposed hit	—	7.60 ± 0.21	1.34 ± 0.44
Palbociclib	Repurposed hit	—	7.69 ± 0.22	1.50 ± 0.51
Imatinib	Repurposed hit	—	2.16 ± 1.47	10.53 ± 1.66

^a^ CNN_VS values for the co‐crystallized ligand and the known inhibitors are single‐structure (static) docking scores from the benchmark dataset (Buccheri and Rescifina 2025); values for the repurposed candidates are the means ± SD across the post‐MD ensemble redocking (Tables S3–S5).

As shown in Table 3, both vemurafenib (7.60 ± 0.21) and palbociclib (7.69 ± 0.22) fell within the activity range of validated SYK inhibitors when evaluated using post‐MD ensemble redocking, with low RMSD values indicating stable binding across the conformational ensembles. In contrast, imatinib, despite a favorable initial docking score, showed a marked decrease in predicted affinity after MD‐based refinement (2.16 ± 1.47) and a substantial increase in RMSD, indicating poor compatibility with the dynamically equilibrated binding site.

Notably, the repurposed candidates were assessed by ensemble redocking, which accounts for protein flexibility and induced‐fit effects and provides a more realistic affinity estimate than the conventional single‐structure docking used in the benchmark set. These findings further support the prioritization of vemurafenib and palbociclib as robust SYK‐targeting candidates for drug repurposing.

### 3.10 Proposed Mechanism of Action and Impact on Inflammatory Signaling

**FIGURE 6:**
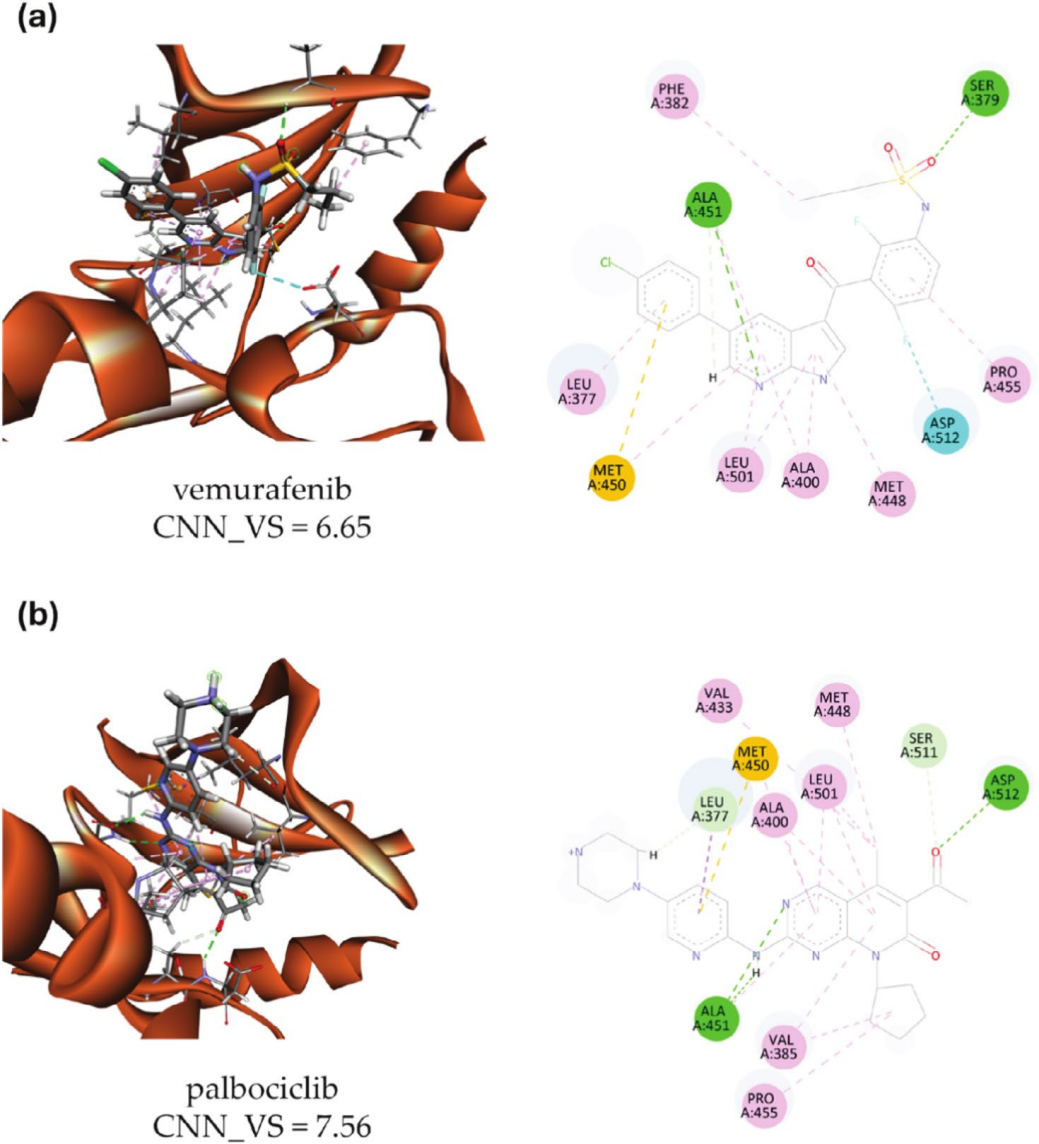
Docked configurations of the ligand–protein complexes. Left: 3D binding mode and molecular information of the ligand. Right: Corresponding 2D interaction network. Panels: (a) vemurafenib, (b) palbociclib.

To visually contextualize the structural basis of the proposed inhibition, the 3D and 2D binding modes of the top two MD‐refined candidates, vemurafenib and palbociclib, are presented in Figure 6. Both compounds effectively occupied the SYK ATP‐binding pocket, maintaining stable interactions with the hinge region and DFG motif, which translated into high predictive‐binding affinities.

Mechanistically, the stable occupancy of this catalytic cleft acts as a competitive block against ATP binding, effectively preventing SYK auto‐phosphorylation and its subsequent activation (Pamuk and Tsokos 2010). In the context of the AT microenvironment, where the TYROBP adaptor protein is aberrantly overexpressed (Sunila et al. 2024), targeted kinase inhibition is crucial. Normally, hyperactive TYROBP recruits and activates SYK, initiating an intracellular cascade that triggers sustained microglial activation (Haure‐Mirande et al. 2022). By dampening SYK catalytic activity at the molecular level, vemurafenib and palbociclib are predicted to interrupt this pathogenic signal‐transduction pathway. Consequently, this pharmacological blockade would translate at the cellular level into a reduced release of neurotoxic factors, including ROS (Zhou et al. 2024) and pro‐inflammatory cytokines, such as TNF‐α and IL‐1β (Haure‐Mirande et al. 2022). Ultimately, providing this structural and mechanistic rationale serves as a vital foundation for the future experimental validation of these repurposed candidates, highlighting how localized kinase inhibition can systemically attenuate microglia‐driven neurodegeneration in AT.

## 4 Conclusions

By integrating structure‐based molecular modeling with network‐driven genomic analysis, this study identified SYK kinase inhibition as a compelling therapeutic strategy for mitigating the neuroinflammatory component of AT. The multilevel computational workflow demonstrated that vemurafenib and palbociclib exhibit structural compatibility, dynamic stability, and network‐level relevance required to effectively engage the SYK catalytic site. Importantly, the incorporation of long‐timescale molecular dynamics and post‐MD redocking enabled the validation of ligand binding in a conformationally relaxed and biologically relevant receptor state, thereby overcoming the limitations inherent to the static docking models.

From a translational perspective, the drug repositioning strategy adopted here offers a substantial advantage for rare diseases, such as AT, where traditional de novo drug development is often impractical owing to time and cost constraints. Although both lead compounds are known substrates of efflux transporters, existing clinical and preclinical evidence from neuro‐oncology indicates that they can achieve pharmacologically meaningful concentrations in the central nervous system. This capability may be further enhanced in AT by localized blood–brain barrier disruption associated with chronic neuroinflammation.

Although the *in silico* evidence presented in this study is robust, the translational feasibility of repurposing potent anticancer agents, such as vemurafenib and palbociclib, for neurodegenerative disorders requires careful experimental validation and toxicity management in vivo. In oncological settings, these drugs are administered at the maximum tolerated dose to achieve cytotoxicity (Aston et al. 2017). However, modulation of neuroinflammatory pathways typically requires significantly lower sub‐oncological doses. This dose reduction could potentially widen the therapeutic window, minimizing systemic toxicity while effectively dampening SYK‐mediated hyperactivation of microglial cells. Furthermore, the well‐characterized safety and pharmacokinetic profiles of these FDA‐approved drugs provide significant advantages for their clinical translation.

To substantiate these computational predictions and move toward clinical applications, a rigorous experimental roadmap is required. Future studies should employ in vitro enzymatic kinase assays to experimentally validate target engagement and determine the precise IC_50_ or *K*_i_ values for SYK inhibition. Subsequently, cell‐based functional assays utilizing AT patient‐derived induced pluripotent stem cells (iPSCs) differentiated into microglia will be essential to evaluate the efficacy of the drugs in suppressing the release of neurotoxic factors, including reactive oxygen species (ROS) and pro‐inflammatory cytokines such as TNF‐α and IL‐1β. The neuroprotective potential should then be assessed in microglial‐neuronal co‐culture systems to evaluate whether drug‐mediated modulation of microglial activation can reduce neuronal injury in a disease‐relevant cellular context. Finally, in vivo validation in ATM‐deficient murine models will be critical to evaluate blood–brain barrier penetrance, establish optimal sub‐toxic dosing regimens, and monitor behavioral and motor phenotype recovery, ultimately confirming the therapeutic viability of targeting the TYROBP‐SYK axis in AT.

Despite the intrinsic limitations of computational screening, this study provides a strong mechanistic and translational rationale for the clinical investigation of SYK‐targeting drugs in AT. Overall, these findings open new avenues for repurposing approved kinase inhibitors as potential neuroprotective agents and may contribute to improving therapeutic options for patients affected by this currently untreatable disorder.

## Author Contributions

**Rocco Buccheri:** investigation, methodology, formal analysis, data curation, writing – original draft, visualization. **Alessia Romano:** investigation, methodology, formal analysis, data curation, writing – original draft. **Antonio Rescifina:** project administration, writing – review and editing, validation, supervision, funding acquisition, conceptualization, methodology. **Chiara Zagni:** conceptualization, validation, writing – review and editing, supervision.

## Funding

This work was supported by the Italian Ministry of Health, Piano di Sviluppo e Coesione del Ministero della Salute 2014–2020, Project: Pharma‐HUB—Hub per il riposizionamento di farmaci nelle malattie rare del sistema nervoso in età pediatrica (CUP E63C22001680001—ID T4‐AN‐04).

## Conflicts of Interest

The authors declare no conflicts of interest.

## Supporting information


**Figure S1:** Per‐residue number of contacts as a function of simulation time for each residue. Panels: (a) vemurafenib, (b) palbociclib, and (c) imatinib.
**Figure S2:** Left: Mean per‐residue SASA (Å^2^) plotted against the residue number, illustrating the preservation of the overall protein folding across all systems. Right: Heatmaps representing the time evolution of the per‐residue SASA over the 200 ns molecular dynamics simulations. The continuous horizontal bands confirm the persistence of stable solvation states without catastrophic unfolding events. Panels: (a) vemurafenib, (b) palbociclib, and (c) imatinib.
**Figure S3:** PCA of the MD trajectories for the ligand‐SYK complexes. Left: PCA eigenvalue spectrum as a function of the eigenvalue number, showing that the first two components (PC1 and PC2) capture most of the conformational variance. Central: Time evolution of the PC1 (blue) and PC2 (orange) projections over the 200 ns simulations. Right: 2D scatter plots of the PC1 vs. PC2 conformational space. Panels: (a) vemurafenib, (b) palbociclib, and (c) imatinib.
**Figure S4:** Time‐dependent MM/PBSA binding energy profiles over 200 ns molecular dynamics trajectories. The background data represent the raw binding energy fluctuations calculated for each extracted snapshot, whereas the overlaid lines denote the moving average (calculated over a 15‐frame window) to highlight the overall thermodynamic trends. Panels: (a) vemurafenib, (b) palbociclib, (c) imatinib, (d) comparison.
**Table S1:** SAveRUNNER output.
**Table S2:** Grid parameters adapted for redocking performed for each cluster. The npts values for each coordinate were 30 and the spacing was 1. The table presents the selected snapshots for each cluster and their corresponding simulation time.
**Table S3:** Redocking performance calculated for all cluster representatives extracted from the MD trajectories. The data show the predicted affinity (CNN_VS) and positional deviation (RMSD) of the redocked poses relative to the corresponding MD conformations of vemurafenib.
**Table S4:** Redocking performance calculated for all cluster representatives extracted from the MD trajectories. The data show the predicted affinity (CNN_VS) and positional deviation (RMSD) of the redocked poses relative to the corresponding MD conformations of palbociclib.
**Table S5:** Redocking performance calculated for all cluster representatives extracted from the MD trajectories. The data show the predicted affinity (CNN_VS) and positional deviation (RMSD) of the redocked poses relative to the corresponding MD conformations of imatinib.
**Table S6:** Average MM/PBSA binding energies calculated over the 200 ns molecular dynamics trajectories.

## Data Availability

The data supporting the findings of this study are available at https://github.com/rocco‐b/SYK‐Kinase‐Inhibitors‐Docking‐and‐MD‐data.
