# Supplementary material for: In Silico Drug Repositioning Identifies SYK Kinase Inhibitors as Potential Neuroprotective Agents for Ataxia–Telangiectasia
Source: Chem Biol Drug Des. 2026 Jul 1;108(1):e70354. doi: 10.1111/cbdd.70354 (PMC13322510; doi:10.1111/cbdd.70354)
Supplement: Supplementary file 1 — Figure S1: Per‐residue number of contacts as a function of simulation time for each residue. Panels: (a) vemurafenib, (b) palbociclib, and (c) imatinib. Figure S2: Left: Mean per‐residue SASA (Å2) plotted against the residue number, illustrating the preservation of the overall protein folding across all systems. Right: Heatmaps representing the time evolution of the per‐residue SASA over the 200 ns molecular dynamics simulations. The continuous horizontal bands confirm the persistence of stable solvation states without catastrophic unfolding events. Panels: (a) vemurafenib, (b) palbociclib, and (c) imatinib. Figure S3: PCA of the MD trajectories for the ligand‐SYK complexes. Left: PCA eigenvalue spectrum as a function of the eigenvalue number, showing that the first two components (PC1 and PC2) capture most of the conformational variance. Central: Time evolution of the PC1 (blue) and PC2 (orange) projections over the 200 ns simulations. Right: 2D scatter plots of the PC1 vs. PC2 conformational space. Panels: (a) vemurafenib, (b) palbociclib, and (c) imatinib. Figure S4: Time‐dependent MM/PBSA binding energy profiles over 200 ns molecular dynamics trajectories. The background data represent the raw binding energy fluctuations calculated for each extracted snapshot, whereas the overlaid lines denote the moving average (calculated over a 15‐frame window) to highlight the overall thermodynamic trends. Panels: (a) vemurafenib, (b) palbociclib, (c) imatinib, (d) comparison. Table S1: SAveRUNNER output. Table S2: Grid parameters adapted for redocking performed for each cluster. The npts values for each coordinate were 30 and the spacing was 1. The table presents the selected snapshots for each cluster and their corresponding simulation time. Table S3: Redocking performance calculated for all cluster representatives extracted from the MD trajectories. The data show the predicted affinity (CNN_VS) and positional deviation (RMSD) of the redocked poses relative to the correspo [file CBDD-108-e70354-s001.pdf]

Supporting Information

In Silico Drug Repositioning Identifies SYK Kinase Inhibitors as Potential Neuroprotective Agents for Ataxia–Telangiectasia

Alessia Romano,[†] Rocco Buccheri,[†] Chiara Zagni,* Antonio Rescifina*

Department of Drug and Health Sciences, University of Catania, Viale A. Doria 6, 95125 Catania, Italy

* Correspondence: chiara.zagni@unict.it

† These authors contributed equally to this study.

Table of Contents

Figure S1. Per-residue number of contacts as a function of simulation time for each residue. Panels: (a) vemurafenib, (b) palbociclib, and (c) imatinib S3

Figure S2. Left: Mean per-residue SASA (Å^2) plotted against the residue number, illustrating the preservation of the overall protein folding across all systems. Right: Heatmaps representing the time evolution of the per-residue SASA over the 200 ns molecular dynamics simulations. The continuous horizontal bands confirm the persistence of stable solvation states without catastrophic unfolding events. Panels: (a) vemurafenib, (b) palbociclib, and (c) imatinib........ S4

Figure S3. PCA of the MD trajectories for the ligand-SYK complexes. Left: PCA eigenvalue spectrum as a function of the eigenvalue number, showing that the first two components (PC1 and PC2) capture most of the conformational variance. Central: Time evolution of the PC1 (blue) and PC2 (orange) projections over the 200 ns simulations. Right: 2D scatter plots of the PC1 vs. PC2 conformational space. Panels: (a) vemurafenib, (b) palbociclib, and (c) imatinib........ S5

Figure S4. Time-dependent MM/PBSA binding energy profiles over 200 ns molecular dynamics trajectories. The background data represent the raw binding energy fluctuations calculated for each extracted snapshot, whereas the overlaid lines denote the moving average (calculated over a 15-frame window) to highlight the overall thermodynamic trends. Panels: (a) vemurafenib, (b) palbociclib, (c) imatinib, (d) comparison S6

Table S1. SAveRUNNER output........ S7

Table S2. Grid parameters adapted for redocking performed for each cluster. The npts values for each coordinate were 30 and the spacing was 1. The table presents the selected snapshots for each cluster and their corresponding simulation time S16

Table S3. Redocking performance calculated for all cluster representatives extracted from the MD trajectories. The data show the predicted affinity (CNN_VS) and positional deviation (RMSD) of the redocked poses relative to the corresponding MD conformations of vemurafenib S16

Table S4. Redocking performance calculated for all cluster representatives extracted from the MD trajectories. The data show the predicted affinity (CNN_VS) and positional deviation (RMSD) of the redocked poses relative to the corresponding MD conformations of palbociclib.. S16
Table S5. Redocking performance calculated for all cluster representatives extracted from the MD trajectories. The data show the predicted affinity (CNN_VS) and positional deviation (RMSD) of the redocked poses relative to the corresponding MD conformations of imatinib S17
Table S6. Average MM/PBSA binding energies calculated over the 200 ns molecular dynamics trajectories...... S17

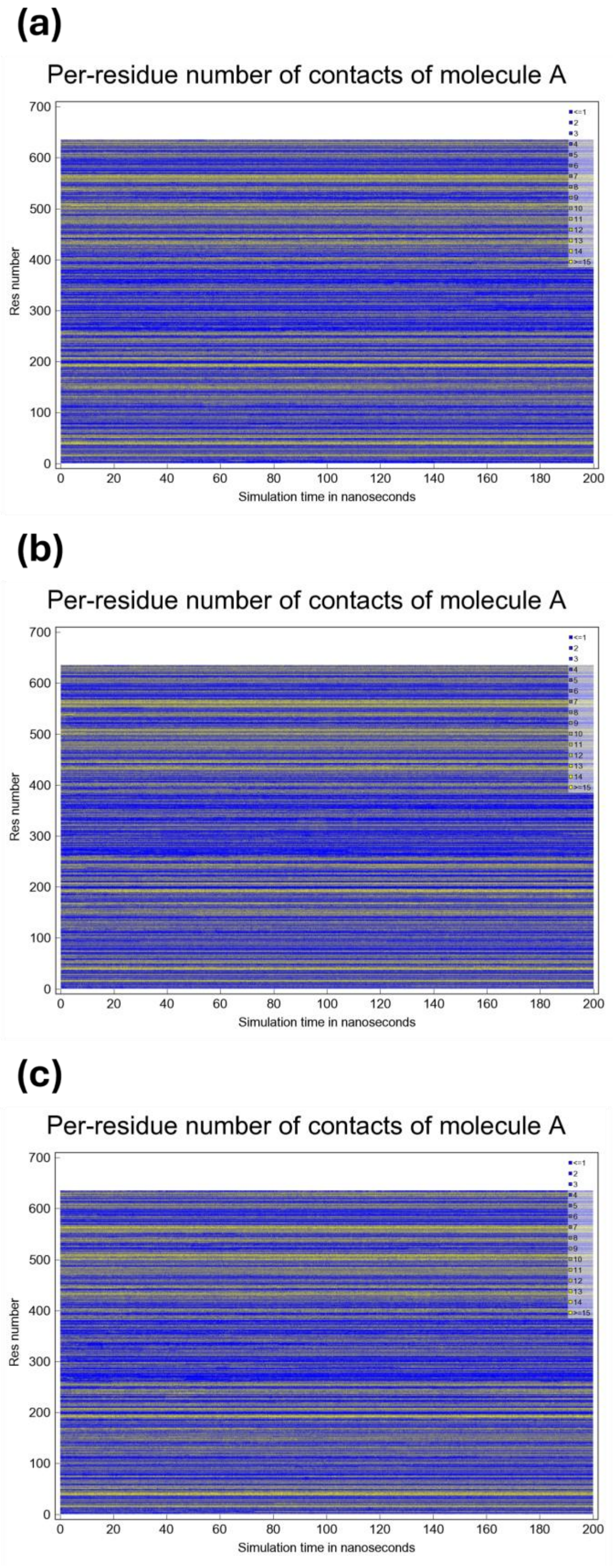

Figure S1. Per-residue number of contacts as a function of simulation time for each residue. Panels: (a) vemurafenib, (b) palbociclib, and (c) imatinib.

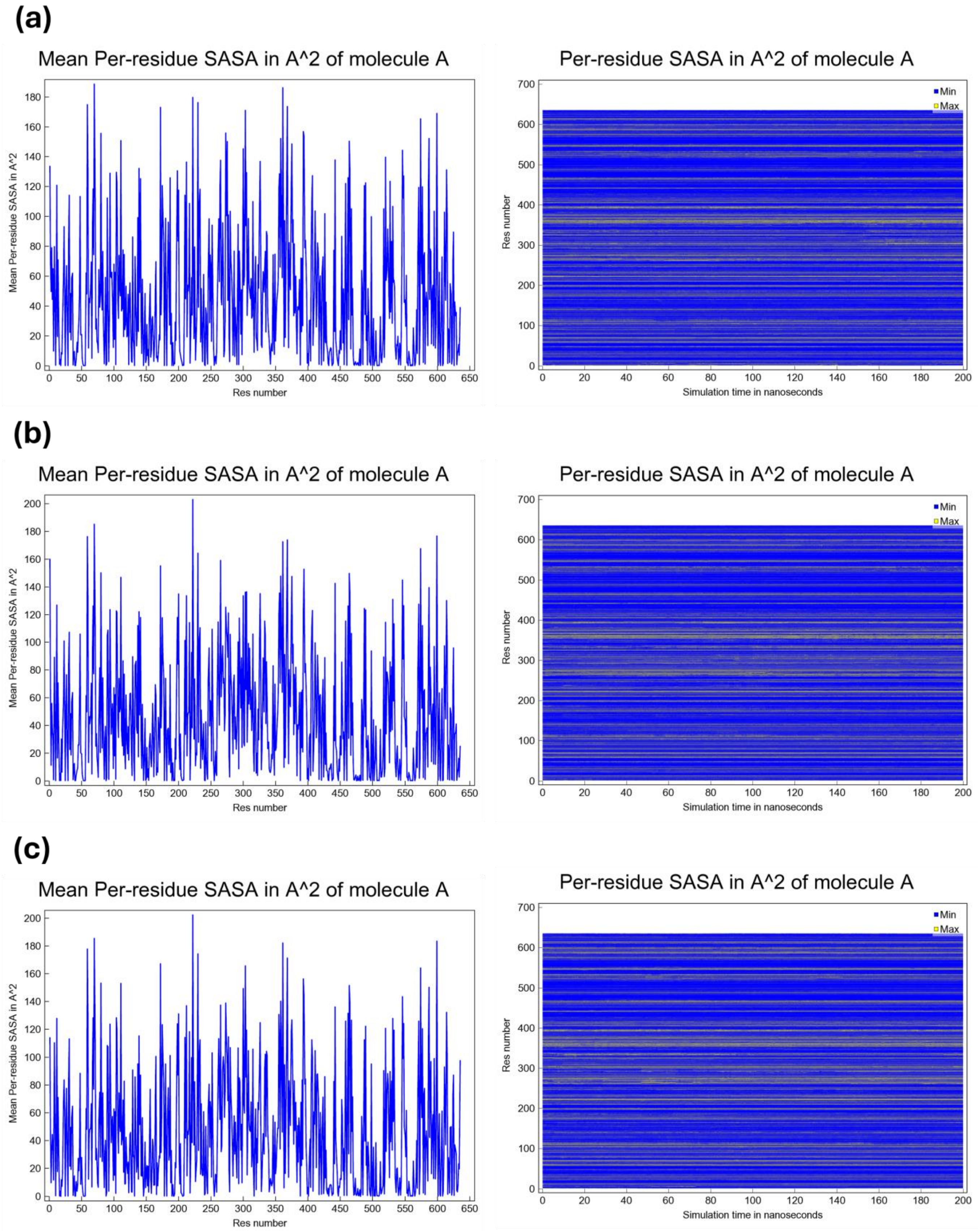

Figure S2. Left: Mean per-residue SASA (Å^2) plotted against the residue number, illustrating the preservation of the overall protein folding across all systems. Right: Heatmaps representing the time evolution of the per-residue SASA over the 200 ns molecular dynamics simulations. The continuous horizontal bands confirm the persistence of stable solvation states without catastrophic unfolding events. Panels: (a) vemurafenib, (b) palbociclib, and (c) imatinib.

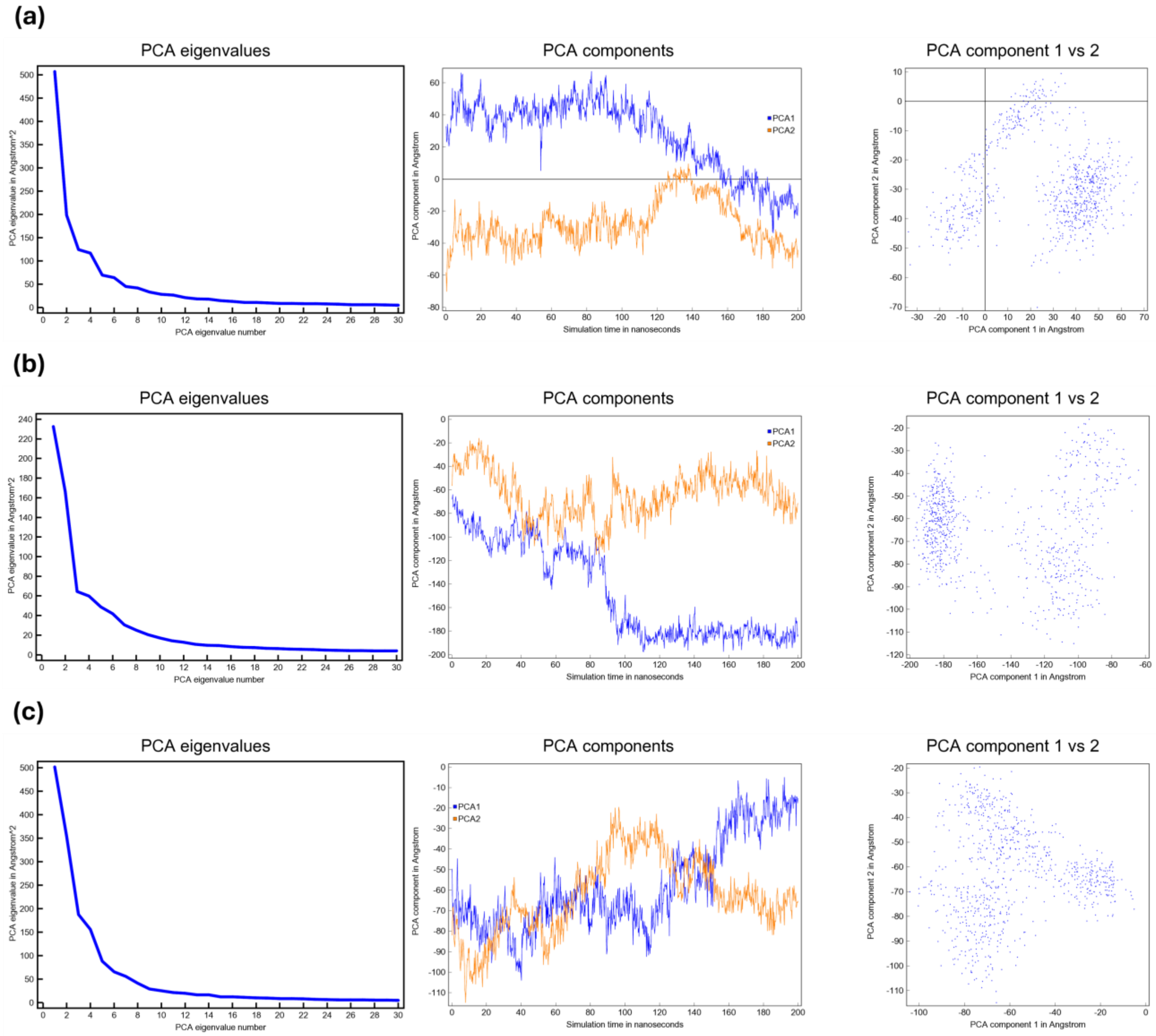

Figure S3. PCA of the MD trajectories for the ligand-SYK complexes. Left: PCA eigenvalue spectrum as a function of the eigenvalue number, showing that the first two components (PC1 and PC2) capture most of the conformational variance. Central: Time evolution of the PC1 (blue) and PC2 (orange) projections over the 200 ns simulations. Right: 2D scatter plots of the PC1 vs. PC2 conformational space. Panels: (a) vemurafenib, (b) palbociclib, and (c) imatinib

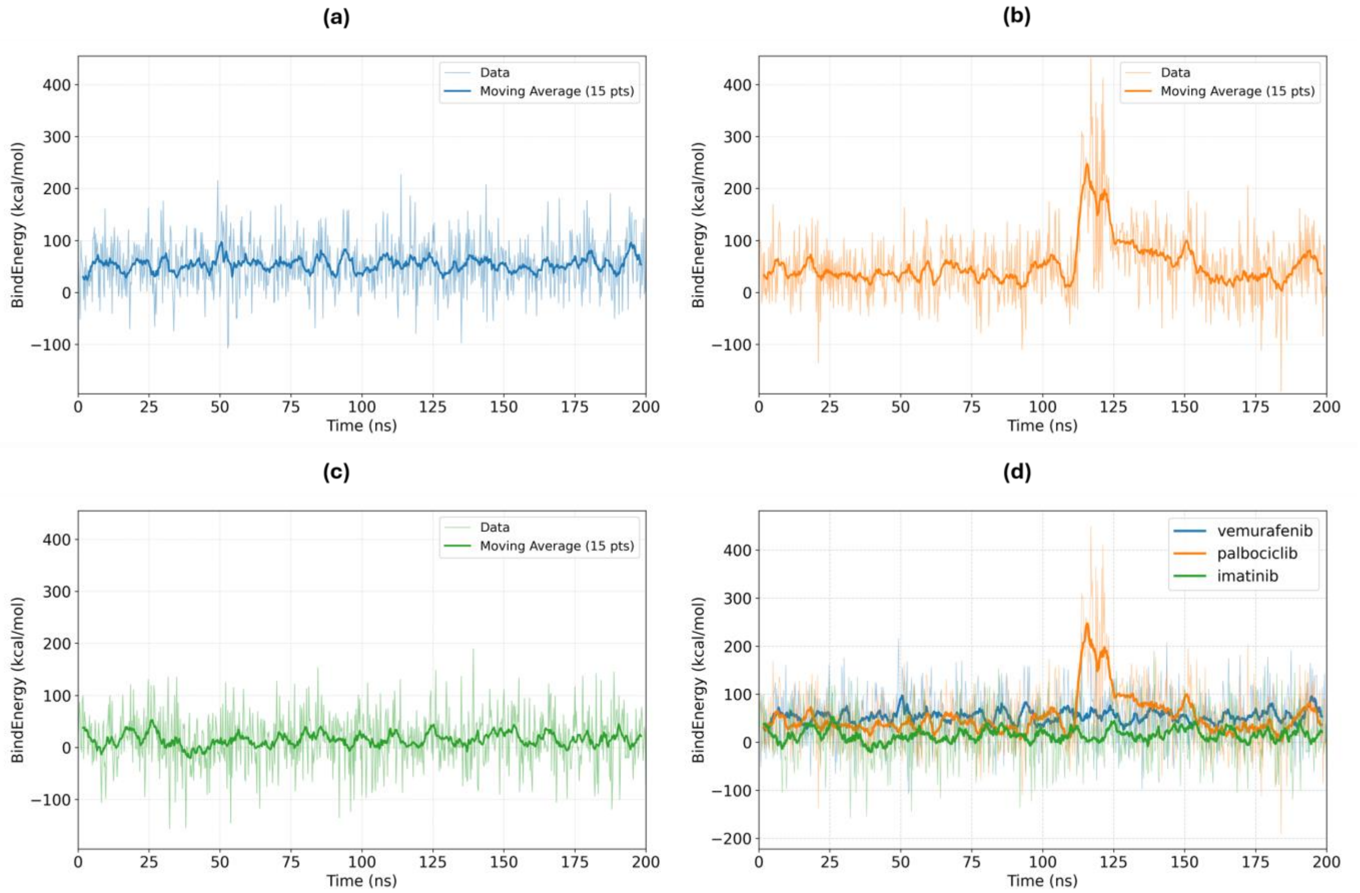

Figure S4. Time-dependent MM/PBSA binding energy profiles over 200 ns molecular dynamics trajectories. The background data represent the raw binding energy fluctuations calculated for each extracted snapshot, whereas the overlaid lines denote the moving average (calculated over a 15-frame window) to highlight the overall thermodynamic trends. Panels: (a) vemurafenib, (b) palbociclib, (c) imatinib, (d) comparison.

Table S1. SAveRUNNER output.

Drug	Proximity	pval	Similarity	Adjusted similarity
5-hydroxytryptophan, iuphar,ligand:4671	0	0.002617	1	0.999954
adagrasib	0	9.74E-05	1	0.999954
adalimumab-adbm, abp 501	0	0.001217	1	0.999954
ado-trastuzumab emtansine, ado-trastuzumab emtansine	0	7.46E-07	1	0.999954
amantadine hydrochloride, amantadine	0	2.76E-08	1	0.999954
amivantamab-vmjw, amivantamab	0	0.006831	1	0.999954
ampicillin trihydrate	0	9.71E-07	1	0.999954
amsacrine, amsacrine	0	5.18E-06	1	0.999954
atezolizumab, mpdl-3280a	0	3.84E-05	1	0.999954
avatrombopag	0	0.000578	1	0.999954
avatrombopag maleate	0	0.000391	1	0.999954
avelumab	0	0.000117	1	0.999954
binimetinib, mek164	0	0.000137	1	0.999954
boceprevir	0	4.79E-07	1	0.999954
cabozantinib s-malate	0	0.000142	1	0.999954
capmatinib	0	0.01131	1	0.999954
capmatinib hydrochloride	0	0.005027	1	0.999954
certolizumab pegol	0	0.002705	1	0.999954
cetuximab, erbitux	0	0.000402	1	0.999954
cinoxacin, cinoxacin	0	0.01401	1	0.999954
cromolyn sodium, sodium cromoglycate	0	6.11E-09	1	0.999954
dabrafenib, gsk 2118436	0	0.001718	1	0.999954
dacarbazine, dtic	0	0.00453	1	0.999954
daunorubicin citrate	0	0.000137	1	0.999954
decitabine, cedazuridine	0	0.001914	1	0.999954
demeclocycline	0	0.00265	1	0.999954
desogestrel, desogestrel	0	0.010385	1	0.999954
dexrazoxane, dexrazoxane	0	3.72E-06	1	0.999954
dostarlimab, dostarlimab-gxly	0	1.27E-10	1	0.999954
doxorubicin liposome, pegylated liposomal doxorubicin	0	4.06E-11	1	0.999954
durvalumab, medi4736	0	2.17E-08	1	0.999954
dydrogesterone, dydrogesterone	0	0.004753	1	0.999954
edaravone, mci-186	0	0.046615	1	0.999954
eltrombopag, eltrombopag olamine	0	0.000112	1	0.999954
encorafenib	0	0.000178	1	0.999954
enoxacin, enoxacin	0	0.000121	1	0.999954
etanercept-szzs, enbrel	0	0.000757	1	0.999954
ethynodiol diacetate, ethynodiol diacetate	0	0.00197	1	0.999954
etoposide phosphate	0	0.000137	1	0.999954
fenretinide, 4-hpr	0	0.000402	1	0.999954
floxuridine, floxuridine	0	0.001163	1	0.999954
glecaprevir	0	0.002157	1	0.999954

golimumab	0	0.000509	1	0.999954
grazoprevir	0	0.000612	1	0.999954
idoxuridine, iudr	0	0.007383	1	0.999954
insulin degludec, insulin degludec	0	0.006378	1	0.999954
insulin susp isophane recombinant human, insulin susp isophane recombinant human	0	0.003518	1	0.999954
insulin susp protamine zinc beef/pork, insulin susp protamine zinc beef/pork	0	0.016923	1	0.999954
insulin zinc susp recombinant human, insulin zinc susp recombinant human	0	0.003499	1	0.999954
inulin, inulin	0	0.003467	1	0.999954
ivosidenib	0	0.003357	1	0.999954
lomustine, ccnu	0	0.005121	1	0.999954
lusutrombopag	0	0.002708	1	0.999954
meropenem anhydrous, meropenem	0	0.00065	1	0.999954
metyrosine, racemetirosine	0	0.004841	1	0.999954
mitoxantrone hydrochloride	0	0.000274	1	0.999954
neratinib, hki-272	0	0.0174	1	0.999954
niclosamide, niclosamide	0	0.034339	1	0.999954
niraparib tosylate	0	0.015515	1	0.999954
niraparib, mk-4827	0	0.027189	1	0.999954
nirogacestat, nirogacestat	0	0.046615	1	0.999954
nomegestrol	0	0.014932	1	0.999954
norgestimate, norgestimate	0	0.012216	1	0.999954
olaparib, azd 2281	0	0.009519	1	0.999954
olutasidenib	0	0.003049	1	0.999954
onapristone	0	0.012216	1	0.999954
osimertinib	0	0.010394	1	0.999954
paritaprevir	0	0.000826	1	0.999954
pefloxacin, pefloxacin	0	0.000402	1	0.999954
pentoxifylline, pentoxifylline	0	0.00011	1	0.999954
pirfenidone, pirfenidone	0	0.002405	1	0.999954
pixantrone	0	0.002157	1	0.999954
podofilox, podofilox	0	9.74E-05	1	0.999954
pralsetinib, blu-667	0	0.003467	1	0.999954
progestogen	0	0.004058	1	0.999954
ramucirumab, imc-1121b	0	0.005476	1	0.999954
retifanlimab, retifanlimab	0	1.70E-07	1	0.999954
ritlecitinib, ritlecitinib	0	5.46E-07	1	0.999954
romiplostim	0	0.000205	1	0.999954
rucaparib camsylate	0	1.25E-05	1	0.999954
segesterone acetate	0	0.002281	1	0.999954
selpercatinib, loxo-292	0	0.01131	1	0.999954
simeprevir sodium	0	0.000298	1	0.999954
simvastatin, simvastin	0	0.000509	1	0.999954
sotorasib	0	0.003138	1	0.999954

sunitinib, su11248	0	0.02664	1	0.999954
talazoparib tosylate	0	0.018743	1	0.999954
talazoparib, 1207456-01-6	0	0.014199	1	0.999954
telaprevir	0	0.000137	1	0.999954
temozolomide, temolozomide	0	0.00197	1	0.999954
tepotinib	0	0.023134	1	0.999954
tepotinib hydrochloride	0	0.016849	1	0.999954
tinidazole	0	0.002708	1	0.999954
trametinib dimethyl sulfoxide, gsk1120212	0	5.46E-07	1	0.999954
triacetin	0	0.001217	1	0.999954
ulipristal acetate	0	0.028911	1	0.999954
valrubicin, valrubicin	0	0.001002	1	0.999954
vemurafenib, zelboraf	0	5.18E-06	1	0.999954
venetoclax, gdc-0199	0	0.038112	1	0.999954
voxilaprevir	0	0.001739	1	0.999954
fluorescein	0.166667	2.50E-07	0.958333	0.999894
indocyanine green acid form, indocyanine green sodium	0.166667	1.60E-09	0.958333	0.999894
temsirolimus, temsirolimus	0.166667	2.70E-07	0.958333	0.999894
panitumumab	0.2	5.09E-14	0.95	0.999875
avobenzone	0.25	4.33E-06	0.9375	0.999839
capivasertib, capivasertib	0.25	0.000515	0.9375	0.999839
diosmin	0.25	3.85E-06	0.9375	0.999839
hydroxystilbamidine isethionate	0.25	0.000222	0.9375	0.999839
tremelimumab, tremelimumab	0.25	9.82E-09	0.9375	0.999839
enzalutamide, enzalutamide	0.272727	2.94E-08	0.931818	0.99982
sapanisertib, sapanisertib	0.285714	0.000102	0.928571	0.999808
cefotaxime sodium	0.3	4.41E-14	0.925	0.999794
alpelisib	0.333333	6.40E-05	0.916667	0.999756
imidocarb dipropionate	0.333333	0.003698	0.916667	0.999756
levonorgestrel, levonorgestrel	0.333333	0.008427	0.916667	0.999756
proflavine	0.333333	0.005255	0.916667	0.999756
vorinostat, vorinostat	0.333333	1.38E-05	0.916667	0.999756
ipilimumab, ipilimumab	0.352941	1.42E-11	0.911765	0.999731
nivolumab, nivolumab	0.375	7.47E-15	0.90625	0.9997
idelalisib, idelalisib	0.4	0.006059	0.9	0.99966
palbociclib, palbociclib	0.4	1.86E-06	0.9	0.99966
toripalimab-tpzi, toripalimab-tpzi	0.4	4.80E-06	0.9	0.99966
vandetanib, vandetanib	0.4	5.08E-05	0.9	0.99966
ribociclib, ribociclib	0.416667	9.12E-06	0.895833	0.999631
pembrolizumab, pembrolizumab	0.428571	1.13E-18	0.892857	0.999608
cobimetinib, cobimetinib	0.454545	2.82E-09	0.886364	0.999554
abemaciclib, abemaciclib	0.461538	4.19E-07	0.884615	0.999538
rucaparib	0.461538	6.94E-05	0.884615	0.999538
regorafenib, regorafenib	0.470588	3.49E-09	0.882353	0.999517
afatinib	0.5	0.007639	0.875	0.999441

benzyl salicylate	0.5	0.007837	0.875	0.999441
capecitabine	0.5	0.006532	0.875	0.999441
carboplatin, paraplatin	0.5	0.002912	0.875	0.999441
carfilzomib, carfilzomib	0.5	0.019535	0.875	0.999441
carminic acid	0.5	1.89E-05	0.875	0.999441
catechin, cianidanol	0.5	5.73E-06	0.875	0.999441
cemiplimab-rwlc, cemiplimab-rwlc	0.5	0.000267	0.875	0.999441
chlormadinone acetate	0.5	0.004954	0.875	0.999441
cobimetinib fumarate	0.5	0.020228	0.875	0.999441
copanlisib, copanlisib	0.5	0.01205	0.875	0.999441
cytarabine, cytosine arabinoside	0.5	0.002025	0.875	0.999441
denosumab, denosumab	0.5	0.01124	0.875	0.999441
dienogest	0.5	0.009016	0.875	0.999441
entacapone	0.5	0.010475	0.875	0.999441
ethambutol hydrochloride, ethambutol	0.5	0.019332	0.875	0.999441
etonogestrel, etonogestrel	0.5	0.032587	0.875	0.999441
fenoterol, fenoterol	0.5	4.13E-06	0.875	0.999441
fruquintinib, fruquintinib	0.5	0.000248	0.875	0.999441
gentian violet cation, crystal violet	0.5	0.000882	0.875	0.999441
gestodene	0.5	0.001418	0.875	0.999441
interferon alfa-2b, ifn alpha-2b	0.5	4.84E-05	0.875	0.999441
lenalidomide, lenalidomide	0.5	0.013678	0.875	0.999441
mefloquine hydrochloride	0.5	0.008889	0.875	0.999441
megestrol acetate, megestrol	0.5	0.008929	0.875	0.999441
miltefosine	0.5	0.007713	0.875	0.999441
nalidixic acid, nalidixic acid	0.5	0.008659	0.875	0.999441
norethindrone acetate	0.5	0.005027	0.875	0.999441
norethynodrel	0.5	0.013472	0.875	0.999441
panobinostat, panobinostat	0.5	5.66E-05	0.875	0.999441
pemetrexed disodium, pemetrexed disodium	0.5	0.000186	0.875	0.999441
procarbazine hydrochloride, pcb	0.5	0.006219	0.875	0.999441
pyrazinamide, pyrazinamide	0.5	0.00585	0.875	0.999441
rofecoxib, vioxx	0.5	0.043968	0.875	0.999441
selumetinib sulfate	0.5	0.021908	0.875	0.999441
sorafenib, bay 43-9006	0.5	0.014479	0.875	0.999441
stavudine, stavudine	0.5	0.000826	0.875	0.999441
testosterone, testosterone undecanoate	0.5	9.92E-13	0.875	0.999441
tislelizumab, tislelizumab	0.5	1.59E-06	0.875	0.999441
tucatinib, tucatinib	0.5	0.024734	0.875	0.999441
urokinase, urokinase-type plasminogen activator	0.5	0.023134	0.875	0.999441
vinorelbine	0.5	0.000121	0.875	0.999441
fulvestrant, fulvestrant	0.545455	0.008649	0.863636	0.999298
everolimus, everolimus	0.555556	3.63E-07	0.861111	0.999262
epirubicin hydrochloride	0.571429	2.98E-05	0.857143	0.999201
methacycline hydrochloride	0.571429	2.22E-08	0.857143	0.999201

rituximab, pertuzumab	0.571429	0.003273	0.857143	0.999201
selumetinib, selumetinib	0.578947	1.35E-08	0.855263	0.999171
gemcitabine	0.590909	7.46E-09	0.852273	0.99912
cladribine, 2-cda	0.6	0.002445	0.85	0.999079
teniposide	0.6	0.048627	0.85	0.999079
tivozanib, tivozanib	0.6	0.001234	0.85	0.999079
trypan blue	0.6	0.020321	0.85	0.999079
sirolimus, sirolimus	0.611111	7.33E-05	0.847222	0.999026
cephapirin sodium	0.615385	1.32E-07	0.846154	0.999005
cefixime anhydrous, cefixime	0.636364	1.15E-05	0.840909	0.998896
letrozole, letrozole	0.636364	0.002723	0.840909	0.998896
mitoxantrone	0.657143	2.60E-13	0.835714	0.998775
allylestrenol	0.666667	0.013472	0.833333	0.998715
baricitinib, baricitinib	0.666667	0.000358	0.833333	0.998715
colchiceine	0.666667	0.021333	0.833333	0.998715
daunorubicin hydrochloride	0.666667	4.34E-11	0.833333	0.998715
erythrosine	0.666667	0.000385	0.833333	0.998715
estradiol valerate, estradiol acetate	0.666667	0.002357	0.833333	0.998715
futibatinib, futibatinib	0.666667	0.004661	0.833333	0.998715
idarubicin	0.666667	1.57E-08	0.833333	0.998715
indigo	0.666667	0.020481	0.833333	0.998715
lenvatinib mesylate	0.666667	0.013268	0.833333	0.998715
methotrexate, tcmdc-125488	0.666667	0.023223	0.833333	0.998715
nintedanib esylate, intedanib	0.666667	0.036327	0.833333	0.998715
norelgestromin	0.666667	0.016709	0.833333	0.998715
raltitrexed, tomudex	0.666667	0.015471	0.833333	0.998715
sacituzumab govitecan, sacituzumab govitecan	0.666667	0.022622	0.833333	0.998715
sulindac, sulindac	0.666667	0.046394	0.833333	0.998715
tazemetostat, tazemetostat	0.666667	0.015489	0.833333	0.998715
tegaserod maleate	0.666667	0.00093	0.833333	0.998715
teprotumumab-trbw, teprotumumab	0.666667	0.005479	0.833333	0.998715
hydroxyzine pamoate	0.7	2.80E-05	0.825	0.998483
lorlatinib, lorlatinib	0.7	0.017701	0.825	0.998483
oxaliplatin, oxaliplatin	0.703704	4.52E-12	0.824074	0.998455
fluphenazine hydrochloride	0.714286	0.013607	0.821429	0.998371
infigratinib, infigratinib	0.714286	0.021537	0.821429	0.998371
lapatinib	0.714286	0.000745	0.821429	0.998371
pentamidine isethionate	0.714286	0.000953	0.821429	0.998371
salmeterol xinafoate	0.714286	0.000629	0.821429	0.998371
estramustine	0.727273	0.000734	0.818182	0.998262
cefuroxime sodium	0.75	0.013754	0.8125	0.998054
crizotinib, crizotinib	0.75	0.004425	0.8125	0.998054
eribulin mesylate, eribulin	0.75	0.002747	0.8125	0.998054
fluocinolone acetonide	0.75	0.014057	0.8125	0.998054
iodoquinol	0.75	0.010385	0.8125	0.998054

isotretinoin, 13-cis-retinoic acid	0.75	0.018225	0.8125	0.998054
luspatercept-aamt, luspatercept	0.75	0.011528	0.8125	0.998054
midostaurin, pkc412	0.75	0.022174	0.8125	0.998054
minocycline hydrochloride	0.75	0.000492	0.8125	0.998054
oxytetracycline anhydrous, oxytetracycline	0.75	2.26E-07	0.8125	0.998054
pacritinib, pacritinib	0.75	0.007834	0.8125	0.998054
romidepsin, romidepsin	0.75	0.015998	0.8125	0.998054
tofacitinib, tofacitinib citrate	0.75	0.020515	0.8125	0.998054
trabectedin, trabectedin	0.75	0.0043	0.8125	0.998054
upadacitinib	0.75	0.028717	0.8125	0.998054
doxorubicin hydrochloride	0.771429	1.06E-07	0.807143	0.997835
azacitidine, azacitidine	0.785714	0.001732	0.803571	0.997675
fludarabine, fludarabine phosphate	0.785714	9.78E-07	0.803571	0.997675
amifostine anhydrous, amifostine	0.8	0.041699	0.8	0.997504
bosutinib, bosutinib	0.8	0.025067	0.8	0.997504
lenvatinib	0.8	0.015719	0.8	0.997504
montelukast sodium	0.8	0.000985	0.8	0.997504
pazopanib, pazopani	0.8	0.025808	0.8	0.997504
quinestrol	0.8	0.032389	0.8	0.997504
tretinoin, all-trans retinoic acid	0.8	0.006714	0.8	0.997504
benzethonium chloride	0.8125	0.027944	0.796875	0.997343
clofarabine	0.8125	1.83E-05	0.796875	0.997343
dopamine	0.818182	0.004753	0.795455	0.997267
amcinonide	0.833333	0.018853	0.791667	0.997053
cefotiam hydrochloride	0.833333	0.002442	0.791667	0.997053
fenoldopam mesylate	0.833333	0.010124	0.791667	0.997053
ceritinib, ceritinib	0.857143	0.043205	0.785714	0.996682
demeclocycline hydrochloride	0.857143	0.003563	0.785714	0.996682
fluorouracil	0.866667	0.001611	0.783333	0.996521
ponatinib	0.866667	0.018906	0.783333	0.996521
topotecan hydrochloride, topotecan	0.866667	0.012246	0.783333	0.996521
4-aminophenol	0.875	0.021968	0.78125	0.996374
rabeprazole, e-3810	0.875	0.031169	0.78125	0.996374
trimetrexate	0.875	0.00606	0.78125	0.996374
camptothecin	0.878788	2.45E-06	0.780303	0.996305
cisplatin, cisplatin	0.87931	3.43E-12	0.780172	0.996296
tannic acid	0.888889	0.037877	0.777778	0.996115
thonzonium bromide	0.888889	0.010784	0.777778	0.996115
erlotinib	0.894737	0.000298	0.776316	0.996
gefitinib, gefitinib	0.9	0.02029	0.775	0.995894
axitinib, axitinib	0.916667	8.12E-05	0.770833	0.995539
inositol	0.928571	0.003744	0.767857	0.995268
methylprednisolone	0.928571	0.007553	0.767857	0.995268
paclitaxel	0.948276	0.00018	0.762931	0.994781
levodopa	0.954545	9.37E-05	0.761364	0.994616

dasatinib anhydrous, dasatinib	0.972603	0.000672	0.756849	0.994111
abatacept	1	0.012547	0.75	0.993254
acetylcysteine, acetylcysteine	1	0.046615	0.75	0.993254
alefacept, alefacept	1	0.034146	0.75	0.993254
allopurinol sodium	1	0.016709	0.75	0.993254
arsenic trioxide, arsenic trioxide	1	0.049755	0.75	0.993254
belatacept	1	0.004446	0.75	0.993254
bendazac	1	0.019817	0.75	0.993254
besifloxacin hydrochloride	1	0.046615	0.75	0.993254
bismuth subsalicylate	1	0.039324	0.75	0.993254
capreomycin sulfate	1	0.029802	0.75	0.993254
cefotetan	1	0.002623	0.75	0.993254
cephalothin sodium	1	0.020255	0.75	0.993254
chloramphenicol palmitate	1	0.030316	0.75	0.993254
chloramphenicol sodium succinate	1	0.013956	0.75	0.993254
ciprofloxacin hydrochloride	1	0.034339	0.75	0.993254
clindamycin hydrochloride	1	0.016709	0.75	0.993254
clindamycin, clindamycin palmitate hydrochloride	1	0.006502	0.75	0.993254
dalfopristin	1	0.011196	0.75	0.993254
diacetylrhein, diacerein	1	0.043407	0.75	0.993254
dihydrostreptomycin sulfate	1	0.031681	0.75	0.993254
dimercaprol	1	0.011184	0.75	0.993254
dirithromycin	1	0.02664	0.75	0.993254
eravacycline dihydrochloride	1	0.003059	0.75	0.993254
erythromycin estolate	1	0.006831	0.75	0.993254
erythromycin gluceptate	1	0.008829	0.75	0.993254
erythromycin lactobionate	1	0.006831	0.75	0.993254
erythromycin stearate	1	0.006744	0.75	0.993254
erythromycin, erythromycin ethylsuccinate	1	0.002281	0.75	0.993254
ezetimibe	1	0.029657	0.75	0.993254
febuxostat	1	0.038112	0.75	0.993254
fosdenopterin, cyclic pyranopterin monophosphate	1	0.026813	0.75	0.993254
fumaric acid, fumaric acid	1	0.001418	0.75	0.993254
gallium nitrate	1	0.006358	0.75	0.993254
galsulfase, galsulfase	1	0.008198	0.75	0.993254
gentamicin sulfate	1	0.004954	0.75	0.993254
halofantrine hydrochloride	1	0.023117	0.75	0.993254
hydroquinone	1	0.013902	0.75	0.993254
kanamycin sulfate	1	0.008929	0.75	0.993254
lefamulin acetate	1	0.022677	0.75	0.993254
levonordefrin	1	0.007146	0.75	0.993254
levosimendan, levosimendan	1	0.046397	0.75	0.993254
lincomycin	1	0.006744	0.75	0.993254
lincomycin hydrochloride	1	0.029802	0.75	0.993254
linolenic acid	1	0.00014	0.75	0.993254

lornoxicam	1	0.043925	0.75	0.993254
meclocycline sulfosalicylate	1	0.021971	0.75	0.993254
melphalan, melphalan	1	0.032675	0.75	0.993254
methsuximide, methsuximide	1	0.008429	0.75	0.993254
mirabegron	1	0.004058	0.75	0.993254
neomycin sulfate	1	0.016709	0.75	0.993254
netilmicin sulfate	1	0.004954	0.75	0.993254
nitisinone	1	0.042411	0.75	0.993254
norfloxacin	1	0.042411	0.75	0.993254
omadacycline tosylate	1	0.021971	0.75	0.993254
oxypurinol	1	0.006831	0.75	0.993254
oxytetracycline calcium	1	0.025344	0.75	0.993254
ozenoxacin	1	0.034339	0.75	0.993254
paramethadione, paramethadione	1	0.00453	0.75	0.993254
paromomycin sulfate	1	0.026153	0.75	0.993254
plazomicin sulfate	1	0.008117	0.75	0.993254
plazomicin, plazomicin	1	0.006433	0.75	0.993254
pyrogallol	1	0.002391	0.75	0.993254
quinupristin	1	0.013956	0.75	0.993254
quinupristin/dalfopristin	1	0.008829	0.75	0.993254
rasagiline, rasagiline	1	0.008392	0.75	0.993254
retapamulin	1	0.016849	0.75	0.993254
rimiterol, rimiterol	1	0.039518	0.75	0.993254
ritodrine hydrochloride	1	0.000481	0.75	0.993254
romosozumab	1	0.016317	0.75	0.993254
selegiline hydrochloride	1	0.028572	0.75	0.993254
sipuleucel-t	1	0.001296	0.75	0.993254
sodium pyruvate, sodium pyruvate	1	0.046615	0.75	0.993254
sotagliflozin	1	0.010876	0.75	0.993254
sparfloxacin	1	0.034339	0.75	0.993254
spectinomycin hydrochloride	1	0.016317	0.75	0.993254
spiramycin	1	0.026813	0.75	0.993254
streptomycin sulfate	1	0.029006	0.75	0.993254
tedizolid	1	0.015713	0.75	0.993254
tedizolid phosphate	1	0.013472	0.75	0.993254
telithromycin	1	0.023297	0.75	0.993254
telotristat ethyl, lx1032	1	0.023297	0.75	0.993254
testolactone, testolactone	1	0.006133	0.75	0.993254
testosterone enanthate	1	0.008929	0.75	0.993254
tetracycline hydrochloride	1	0.023297	0.75	0.993254
tetracycline phosphate complex	1	0.019392	0.75	0.993254
tiagabine hydrochloride	1	0.006502	0.75	0.993254
tigecycline	1	0.019392	0.75	0.993254
tobramycin	1	0.026153	0.75	0.993254
toremifene	1	0.003859	0.75	0.993254

troglitazone, troglitazone	1	0.017036	0.75	0.993254
urea	1	0.016798	0.75	0.993254
vibegron	1	0.023262	0.75	0.993254
vinblastine, vinblastine	1	0.023551	0.75	0.993254
viomycin sulfate	1	0.001386	0.75	0.993254
masoprocol, nordihydroguaiaretic acid	1.026316	0.000162	0.743421	0.992314
norepinephrine	1.045455	0.001756	0.738636	0.99155
leucovorin calcium, leucovorin	1.058824	0.029163	0.735294	0.990972
quercetin	1.061224	1.22E-05	0.734694	0.990864
ethacrynic acid	1.0625	0.011419	0.734375	0.990806
thioguanine	1.066667	0.003001	0.733333	0.990615
epoetin alfa, erythropoietin	1.071429	0.049621	0.732143	0.990391
bevacizumab-awwb, bevacizumab	1.078947	0.01828	0.730263	0.990028
tamoxifen, tamoxifen	1.095238	0.012697	0.72619	0.989192
metformin, metformin	1.103448	0.00156	0.724138	0.988745
methylene blue anhydrous, methylene blue	1.105263	0.001313	0.723684	0.988644
digitoxin	1.111111	0.00715	0.722222	0.988311
liothyronine sodium	1.111111	0.020315	0.722222	0.988311
etoposide, etoposide	1.125	0.014389	0.71875	0.987482
thalidomide, thalidomide	1.133333	0.022135	0.716667	0.986958
methyldopa anhydrous, methyldopa	1.137931	0.000431	0.715517	0.986659
imatinib, imatinib	1.147059	0.009746	0.713235	0.986045
zinc sulfate anhydrous, zinc sulfate	1.166667	0.038344	0.708333	0.984633
irinotecan hydrochloride, irinotecan	1.18	0.004112	0.705	0.983592
alendronate sodium, alendronate	1.2	0.003533	0.7	0.981901
enoximone	1.2	0.046501	0.7	0.981901
reserpine	1.217391	0.034447	0.695652	0.980291
norethindrone, norethisterone	1.25	0.043205	0.6875	0.976888
capsaicin	1.266667	0.023776	0.683333	0.974933
daunorubicin liposomal, daunorubicin	1.271605	0.011019	0.682099	0.974323
mercaptopurine	1.277778	0.047986	0.680556	0.973541
ascorbic acid	1.3	0.024037	0.675	0.970528
etravirine	1.333333	0.035052	0.666667	0.965377
selegiline, selegiline	1.333333	0.027189	0.666667	0.965377
ampicillin (anhydrous), ampicillin sodium	0.666667	0.015143	0.833333	0.161014

Table S2. Grid parameters adapted for redocking performed for each cluster. The npts values for each coordinate were 30 and the spacing was 1. The table presents the selected snapshots for each cluster and their corresponding simulation time.

Compound	Cluster	Snapshot	Time (ns)	Center x	Center y	Center z
Vemurafenib	1	80	20.00	34.691	32.646	29.695
Vemurafenib	2	131	32.75	34.055	41.678	25.315
Vemurafenib	3	227	56.75	30.756	39.045	18.409
Vemurafenib	4	329	82.25	29.341	49.294	20.999
Vemurafenib	5	431	107.75	29.620	42.881	18.861
Vemurafenib	6	483	120.75	26.624	42.436	15.282
Vemurafenib	7	609	152.25	3.075	37.922	11.170
Vemurafenib	8	635	158.75	-3.154	42.818	14.602
Vemurafenib	9	675	168.75	-1.571	38.438	14.376
Vemurafenib	10	736	184.00	3.208	30.494	13.654
Palbociclib	1	440	110.00	34.560	22.628	48.323
Palbociclib	2	562	140.52	1.681	20.058	56.244
Palbociclib	3	683	170.75	-3.502	21.407	55.604
Imatinib	1	80	20.00	22.432	33.466	11.302
Imatinib	2	138	34.50	31.186	28.204	20.943
Imatinib	3	169	42.25	21.524	20.816	18.073
Imatinib	4	205	51.25	10.424	17.435	19.179
Imatinib	5	249	62.25	13.974	31.711	9.527
Imatinib	6	296	74.00	5.909	56.942	15.322
Imatinib	7	322	80.50	6.356	53.238	12.536
Imatinib	8	365	91.25	13.324	52.469	11.900
Imatinib	9	524	131.00	-6.192	55.456	24.140
Imatinib	10	588	147.00	11.598	56.602	15.562
Imatinib	11	662	165.50	2.626	55.152	15.519

Table S3. Redocking performance calculated for all cluster representatives extracted from the MD trajectories. The data show the predicted affinity (CNN_VS) and positional deviation (RMSD) of the redocked poses relative to the corresponding MD conformations of vemurafenib.

Cluster	CNN_VS	RMSD (Å)
1	7.42	1.63
2	7.50	0.72
3	7.59	1.21
4	7.62	0.67
5	7.61	1.88
6	8.09	0.89
7	7.78	1.69
8	7.43	1.75
9	7.39	1.49
10	7.61	1.44

Table S4. Redocking performance calculated for all cluster representatives extracted from the MD trajectories. The data show the predicted affinity (CNN_VS) and positional deviation (RMSD) of the redocked poses relative to the corresponding MD conformations of palbociclib.

Cluster	CNN_VS	RMSD (Å)
1	7.63	1.71
2	7.94	1.86
3	7.51	0.92

Table S5. Redocking performance calculated for all cluster representatives extracted from the MD trajectories. The data show the predicted affinity (CNN_VS) and positional deviation (RMSD) of the redocked poses relative to the corresponding MD conformations of imatinib.

Cluster	CNN_VS	RMSD (Å)
1	2.64	11.83
2	1.30	11.70
3	3.44	9.41
4	0.86	9.66
5	0.77	9.69
6	1.55	12.07
7	5.63	10.02
8	3.29	12.04
9	1.24	11.50
10	1.76	6.62
11	1.28	11.32

Table S6. Average MM/PBSA binding energies calculated over the 200 ns molecular dynamics trajectories.

Compound	Binding Energy Avg (kcal/mol)[a]
vemurafenib	53.583
palbociclib	52.448
imatinib	15.218

[a] According to the YASARA energy calculation convention, the binding energy is derived from the potential and solvation energies of the isolated components minus those of the complex. Consequently, positive values denote thermodynamically favorable and stable binding interactions.
